# Efficacy and safety of complement inhibitors in patients with geographic atrophy associated with age-related macular degeneration: a network meta-analysis of randomized controlled trials

**DOI:** 10.3389/fphar.2024.1410172

**Published:** 2024-11-12

**Authors:** Huan Wang, Jiaqi Zheng, Qing Zhang, Zhongping Tian, Yuhang Sun, Tianyi Zhu, Yanlong Bi, Li Zhang

**Affiliations:** ^1^ Clinical Research Center, Tongji Hospital, Tongji University School of Medicine, Shanghai, China; ^2^ Research Unit of Molecular Epidemiology, Helmholtz Zentrum München, Neuherberg, Germany; ^3^ Department of Ophthalmology, Tongji Hospital, Tongji University School of Medicine, Shanghai, China; ^4^ Tongji Eye Institute, Tongji University School of Medicine, Shanghai, China

**Keywords:** complement inhibitors, geographic atrophy, age-related macular degeneration, network meta-analysis, meta-analysis

## Abstract

**Importance:**

Clinical trials in recent years have shown significant effectiveness of complement inhibitors for geographic atrophy (GA) treatment. Two complement inhibitor drugs have been approved by the Food and Drug Administration (FDA).

**Objective:**

to compare and rank the different complement inhibitors in the treatment of GA secondary to age-related macular degeneration (AMD).

**Data sources:**

A systematic literature search was conducted in the Cochrane Central, Web of Science Core Collection, PubMed, LWW Medical Journals, ClinicalTrials.gov, and WHO ICTRP from inception to October 2023.

**Study selection:**

All randomized clinical trials evaluating the effectiveness of complement inhibitors in patients diagnosed with secondary GA in AMD were identified.

**Data extraction and synthesis:**

This study followed Preferred Reporting Items for Systematic reviews and Meta-Analyses (PRISMA) network meta-analysis Checklist of Items and the Cochrane Risk of Bias Assessment Tool for assessing the study quality. Multiple authors independently coded all titles and abstracts, reviewed full-text articles against the inclusion and exclusion criteria, and resolved all discrepancies by consensus. Random-effects network meta-analyses were applied. Bayesian network meta-analysis was performed using the BUGSnet package in R (4.2.0).

**Main outcomes and measures:**

The primary efficacy outcome was the change in GA lesion size (mm^2^) from baseline to month 12. The secondary efficacy outcome was the mean change in best-corrected visual acuity (BCVA) from baseline to month 12. Safety outcome measures included the number of subjects with serious adverse events (SAEs) and macular neovascularization (MNV).

**Results:**

Ten randomized controlled trials including 4,405 participants and five complement inhibitors were identified. Comparison with sham and SUCRA analysis showed that avacincaptad pegol 2 mg (MD: −0.58, 95% CrI: −0.97 to −0.18, SUCRA: 93.55), pegcetacoplan monthly (MD: −0.38, 95% CrI: −0.57 to −0.20, SUCRA: 81.37), and pegcetacoplan every other month (MD: −0.30, 95% CrI: −0.49 to −0.11, SUCRA: 70.16) have significant changes in GA lesion reduction. No treatments showed significant changes in BCVA and SAE compared with sham. Pegcetacoplan monthly (OR: 4.30, 95% CrI: 1.48–16.72) increased the risk of MNV. Avacincaptad pegol 2 mg demonstrated favorable outcomes in terms of SAE and MNV.

**Conclusion and relevance:**

Avacincaptad pegol 2 mg is the most effective complement inhibitor with better safety for the treatment of GA secondary to AMD.

**Systematic Review Registration:**

https://www.crd.york.ac.uk/prospero/display_record.php?ID=CRD42022351515, Identifier PROSPERO CRD42022351515.

## Highlights

• Question: What is the comparative effectiveness and safety of complement inhibitors in improving geographic atrophy (GA) in patients with age-related macular degeneration (AMD)?
• Findings: In this network meta-analysis including 10 trials with 4,405 participants, both avacincaptad pegol and pegcetacoplan caused a reduction in the GA area in AMD patients at 1 year. Indirect comparisons suggest that avacincaptad pegol 2 mg has the potential to be superior to pegcetacoplan and has better safety.
• Meaning: These findings indicate that avacincaptad pegol may be more effective and safe for reducing the progression of GA for patients with AMD.

## 1 Introduction

Age-related macular degeneration (AMD), as the most common form of maculopathy, is a major cause of vision loss in elderly people and shows a steep increase in prevalence after the age of 50 years (Jonas et al., 2017; Wong et al., 2014; Vangsted et al., 2023; Vujosevic et al., 2023). Geographic atrophy (GA) is the advanced stage of AMD, which can lead to progressive and irreversible loss of visual function due to loss of the retinal photoreceptors, retinal pigment epithelium (RPE), and choriocapillaris (Nittala et al., 2022). In the absence of treatment, 66% of eyes with GA may lose vision or become severely visually impaired during a patients’ lifetime (Colijn et al., 2021). It is estimated that the number of cases with late AMD in 2050 would be 6.41 million (95% CrI: 3.37–13.22) worldwide (Wang et al., 2022).

For a long time, there were no effective drugs for treating GA secondary to AMD. The American Academy of Ophthalmology guidelines recommended that patients with advanced AMD consider using antioxidant, vitamin, and mineral supplements (Flaxel et al., 2020). However, recent clinical trials have shown promising advancement in treatment options, including diet therapy, antibody therapy, gene therapy, cell therapy, visual cycle modulators, photobiomodulation, and laser therapy. In February 2023, pegcetacoplan, a complement C3 cyclic peptide inhibitor, was approved by the Food and Drug Administration (FDA) as the first-line treatment to treat GA (Schachar, 2023). More recently, another complement inhibitor avacincaptad pegol, a C5 inhibitor, was also approved by the FDA for the treatment of GA secondary to AMD (Kang, 2023).

Numerous genetic and molecular studies have confirmed the significant role of the complement system in AMD, including genetic variants, overactivation of alternative pathway, inflammation, oxidative stress, lipid accumulation, and energy metabolism (Qin et al., 2021; Riley-Gillis et al., 2023). Potential therapeutic targets within the complement system include C1q, C3, C5, complement factors (B, D, H, and I), as well as membrane attack complex and properdin (Patel et al., 2022; Lambris et al., 1996; Mannes et al., 2020). Several randomized controlled trials (RCTs) have been conducted to evaluate the efficacy of complement inhibitors in treating GA. The aim of this network meta-analysis (NMA) was to compare and rank the different complement inhibitors in the treatment of GA secondary to AMD.

## 2 Methods

### 2.1 Study design and search strategy

This NMA study followed the Preferred Reporting Items for Systematic Reviews and meta-analysis (PRISMA-NMA) guidelines (Hutton et al., 2015) and was registered with PROSPERO (registration number CRD42022351515). A comprehensive literature review was conducted in the Cochrane Central, Web of Science Core Collection, PubMed, LWW Medical Journals, ClinicalTrials.gov, and WHO ICTRP databases to identify eligible publications (up to October 2023). Additionally, we manually searched the references of relevant reviews, systematic reviews, conferences, and included studies to identify other potentially eligible studies. The detailed search strategy is available in Supplementary eAppendix S1, S1.

### 2.2 Eligibility criteria

Participants included patients who were ≥50 years of age and diagnosed with GA secondary to AMD. Sex, race, and source of the case were not limited.

### 2.3 Interventions and comparisons of complement inhibitors and sham

Outcomes: the primary efficacy outcome was change in GA lesion size from baseline to month 12, measured in square millimeters (mm^2^) using fundus autofluorescence (FAF). The secondary efficacy outcome was mean change in best-corrected visual acuity (BCVA), measured in the Early Treatment Diabetic Retinopathy Study (ETDRS) letters, from baseline to month 12. For safety outcomes, we analyzed the number of subjects with serious adverse events (SAEs) and macular neovascularization (MNV) in both eyes. In tallying the occurrences of MNV, macular choroidal neovascularization, neovascular AMD, and exudative AMD were also considered.

Type of study: All published and unpublished RCTs were included. There were no language restrictions, and we did not exclude studies based on the date of publication.

### 2.4 Exclusion criteria

Participants were excluded based on the following criteria (Jonas et al., 2017): causes of GA other than AMD (Wong et al., 2014), eye surgery or intravitreal injection in the eye (Vangsted et al., 2023), trials that were not RCTs (Vujosevic et al., 2023), follow-up time less than 1 year (Nittala et al., 2022), studies with imbalanced or incomparable baseline data between the two groups (Colijn et al., 2021), and studies lacking primary or secondary outcomes or with unextractable data.

### 2.5 Study selection

Citations identified from the literature and reference list searches were imported to EndNote, and duplicates were removed. Two researchers (H W and YH S) independently reviewed titles and abstracts. The researchers (TY Z and ZP T) then independently screened the titles and abstracts of all retrieved articles in pairs. In cases of disagreement, consensus on which articles to screen for full text was reached by discussion. If necessary, a third researcher (L Z) was consulted to make a final decision. After this, two researchers (JQ Z and Q Z) independently screened the full-text articles for inclusion. Again, in cases of disagreement, a consensus on inclusion or exclusion was reached by discussion, and if necessary, a third researcher was consulted (YL B).

### 2.6 Data extraction and synthesis

Data for NMA were extracted using a custom-made Excel worksheet. Two investigators (H W and JQ Z) independently extracted data from the studies. Data of the following items were extracted (Jonas et al., 2017): study characteristics: first author, year of publication, region, number of patients, study design, trial phase, drug doses and frequency, follow-up duration, and inclusion/exclusion criteria (Wong et al., 2014); patient characteristics: age, disease duration, and disease severity at baseline (Vangsted et al., 2023); primary outcomes: mean change in GA from baseline to month 12 (Vujosevic et al., 2023); secondary outcomes: mean change in BCVA from baseline to month 12 (Nittala et al., 2022); safety outcomes: number of SAEs and MNV events.

The evaluation results were represented using a literature quality evaluation chart. Data extraction and risk of bias assessment were independently completed and cross-checked by multiple researchers. The Cochrane Collaboration’s tool (Higgins, 2011) was used to assess the risk of bias of included RCTs. Data were analyzed using a random-effects model.

### 2.7 Network meta-analysis

The BUGSnet (Bayesian inference Using Gibbs Sampling to conduct network meta-analysis) package on R (Béliveau et al., 2019) conducts Bayesian NMA in compliance with the best practice and reporting guidelines. The network diagram of each outcome was drawn to visualize the connections between different interventions. All NMA models were based on a Bayesian approach through the Markov Chain Monte Carlo (MCMC) simulation. The parameters assessed in the NMA models were treatment effects compared to other treatment arms, and the likelihood function was dependent on the outcome (Schweighoffer et al., 2022). The Gelman–Rubin–Brooks plot was used to identify potential outliers and determine the optimal effect model; adequacy of the model fit was assessed through a comparison of the residual deviance of the models, where the lower the value of the deviance information criterion (DIC), the better the fitting effect of the model. We construct convergence diagnostic graphs and trajectory density graphs to test the convergence and stability of the model, with a potential scale reduction factor (PSRF) value of <1.05 considered an indication that the simulation is valid. The global inconsistency detection method was used for consistency test, plotting the posterior mean deviance of a consistency model vs. an inconsistency model (unrelated mean effect model, NME). Paired direct comparison and Higgins and Thompson’s I^2^ statistic tests were used for checking the homogeneity assumption. The mean GA change and BCVA were pooled as mean difference (MD) with posterior median and 95% credible intervals (CrI). SAE and MNV were pooled as odds ratio (OR) with 95% CrI. Surface Under the Cumulative Ranking Curve (SUCRA) analysis was performed to rank treatment arms according to their efficacy. The SUCRA plot and score are presented in the *Results.* All analyses were conducted using R software (version 4.2.0, R project; with packages BUGSnet_v1.1.0, robvis_v0.3.0, dplyr_v1.1.2, and tidyr_v1.2.0), and JAGS (version_v4.3.1, http://mcmc-jags.sourceforge.net). The functions of the BUGSnet package are shown in Supplementary eAppendix S2, S1.

## 3 Results

### 3.1 Study identification and selection

**FIGURE 1:**
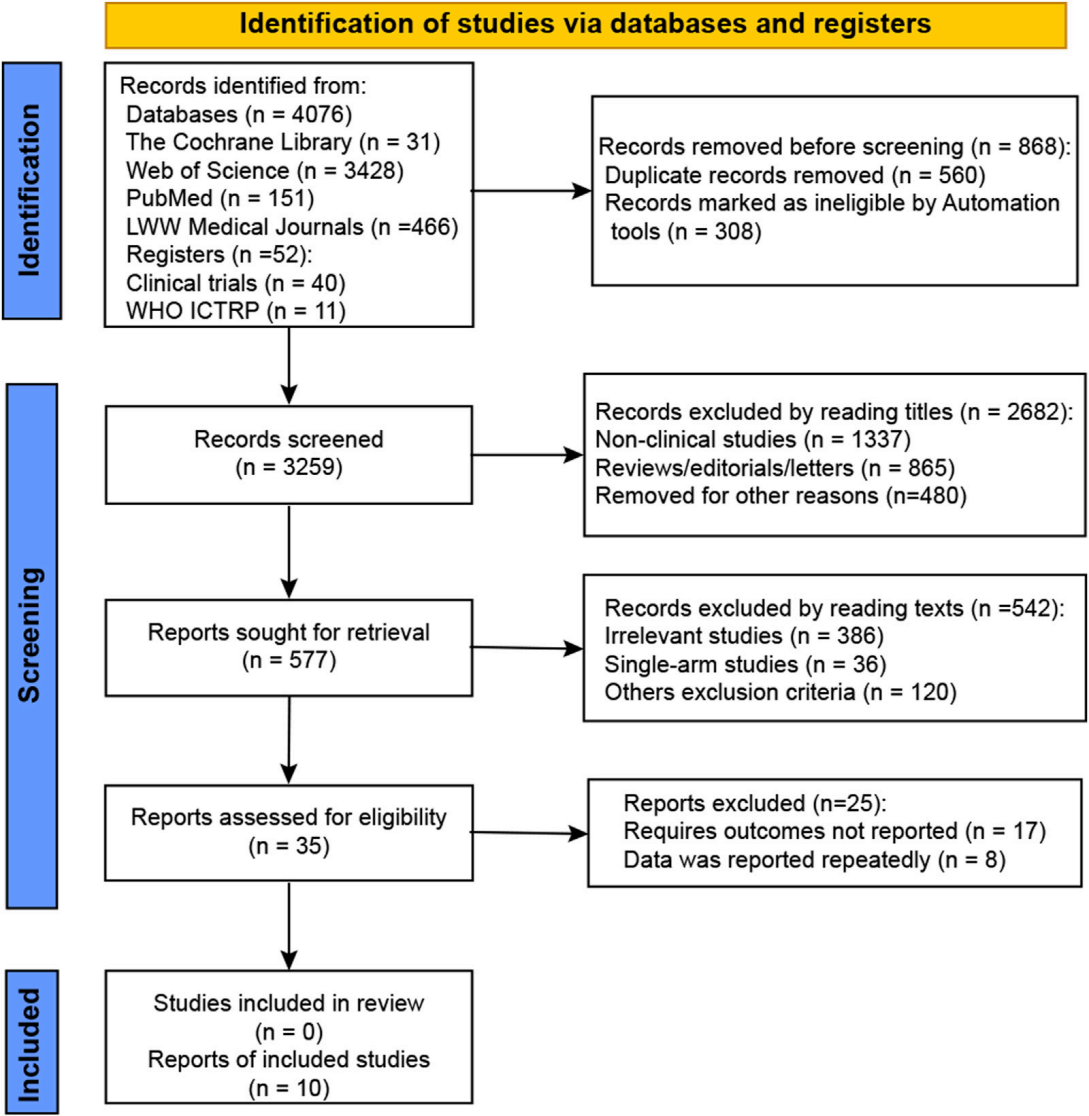
Flow diagram of study selection.

We retrieved 4,128 studies from the electronic databases and as a result of manual search, as shown in Figure 1. After removing duplicates, 3,259 records were screened for eligibility. Of these, 2,682 records were excluded based on title review, and 577 records were excluded after full-text review. Eventually, 10 studies that evaluated intravitreal administrations against sham were included in this meta-analysis (NCT02515942, NCT01527500, NCT02686658, NCT04435366, NCT02503332, NCT03525613, NCT03525600, NCT01229215, NCT02247479, and NCT02247531).

### 3.2 Characteristics of included studies

The study characteristics of the final 10 RCTs are listed in Table 1 (Jaffe et al., 2021; Khanani et al., 2023; Liao et al., 2020; Heier et al., 2023; Yaspan et al., 2017; Holz et al., 2018); they were all registered on ClinicalTrials.gov. A total of 4,405 patients with GA secondary to AMD were included in this NMA. The average age of the patients ranged from 76.3 to 77.7 years, and the proportion of female patients varied from 36.4% to 71.8%. Each study included a control group administered sham intravitreal injection. Five different complement inhibitors were identified: pegcetacoplan, avacincaptad pegol, lampalizumab, CLG561, and LFG316. All studies had a follow-up period of over 1 year. Data from all studies are available on ClinicalTrials.gov or have been published in PubMed.

**TABLE 1:** Overview of included studies.

Study	Year	Target	Phase	sample	Interventions	Age	Female (%)	Primary outcome measure
NCT02515942	2018	Properdin	II	114	CLG561	77.7 (8.6)	22 (61.1)	Percentage of SAE and change in GA lesion size measured by FAF from baseline to day 337
					CLG561 & LFG316	78.8 (7.1)	21 (53.6)	
					Sham	78.7 (9.8)	24 (61.5)	
NCT01527500	2018	C5	II	158	LFG316	78.6 (7.5)	58 (58.6)	Change in GA lesion size measured by FAF from baseline to day 505
					Sham	80.8 (6.5)	32 (62.8)	
NCT02686658 (Jaffe et al., 2021)	2021	C5	II/III	286	Avacincaptad pegol 2 mg	78.8 (10.2)	45 (67.2)	Change in GA lesion size measured by FAF from baseline to month 12
					Sham	78.2 (8.8)	79 (71.8)	
					Avacincaptad pegol 4 mg	79.2 (8.3)	58 (69.9)	
					Sham	78.2 (9.0)	61 (72.6)	
NCT04435366 (Khanani et al., 2023)	2023	C5	III	447	Avacincaptad pegol 2 mg	76.3 (8.6)	154 (68.4)	Mean rate of change in GA over 12 months
					Sham	76.7 (8.8)	156 (70.3)	
NCT02503332 (Liao et al., 2020)	2019	C3	II	246	Pegcetacoplan monthly	79.6 (7.51)	55 (63.9)	Least square mean change from baseline in square root GA lesion size in the study eye at month 12
					Pegcetacoplan EOM	81.0 (7.55)	50 (63.3)	
					Sham	78.4 (7.43)	49 (60.5)	
NCT03525613 (Heier et al., 2023)	2023	C3	III	613	Pegcetacoplan monthly	79.0 (7.21)	134 (62.9)	Change from baseline to month 12 in the total area of GA lesion(s) in the study eye based on FAF
					Pegcetacoplan EOM	78.1 (7.81)	122 (57.5)	
					Sham	78.1 (7.81)	136 (64.2)	
NCT03525600 (Heier et al., 2023)	2023	C3	III	621	Pegcetacoplan monthly	78.8 (6.91)	85 (41.3)	Least square mean change from baseline in total area of GA lesions in the study eye at month 12
					Pegcetacoplan EOM	79.2 (7.06)	82 (39.4)	
					Sham	78.5 (7.24)	75 (36.4)	
NCT01229215 (Yaspan et al., 2017)	2017	Factor D	Ib/II	123	Lampalizumab monthly	80.4 (7.2)	28 (65.1)	Growth rate of geographic atrophy (GA) lesion area from baseline to month 12
					Lampalizumab EOM	80.4 (7.2)	18 (40.9)	
					Sham	78.5 (7.3)	24 (57.1)	
NCT02247479 (Holz et al., 2018)	2018	Factor D	III	858	Lampalizumab monthly	77.5 (7.9)	182 (61.1)	Change in the GA area, as assessed by retinal imaging from baseline to month 12
					Lampalizumab EOM	78.3 (8.5)	185 (61.1)	
					Sham	78.5 (7.8)	186 (61.0)	
NCT02247531 (Holz et al., 2018)	2018	Factor D	III	939	Lampalizumab monthly	77.3 (7.9)	197 (59.7)	Change in the GA area, as assessed by retinal imaging from baseline to month 12
					Lampalizumab EOM	78.7 (8.0)	190 (58.6)	
					Sham	77.6 (8.3)	191 (59.5)	

### 3.3 Risk of bias in included studies

All studies included in the analysis adopted a multicenter, international, randomized, parallel-group design with comparator groups and completed the planned follow-up. Nine studies used web-based randomization systems to generate random sequences, which were either completely randomized, block-randomized, or dynamically miniaturized, with central randomization concealment. One study did not specify the methods for generating random sequences or the masking procedure. Six studies implemented double masking, and all ten studies used blinded methods for assessing outcome measures. All studies reported complete data, though two had a dropout rate exceeding 20%. The intention-to-treat analysis was applied across all studies, and no selective reporting of research outcomes was identified. All included studies were classified as having low risk. The risk of bias assessment is summarized in Supplementary eAppendix S3, S1.

## 4 Results of network relationship analysis

**FIGURE 2:**
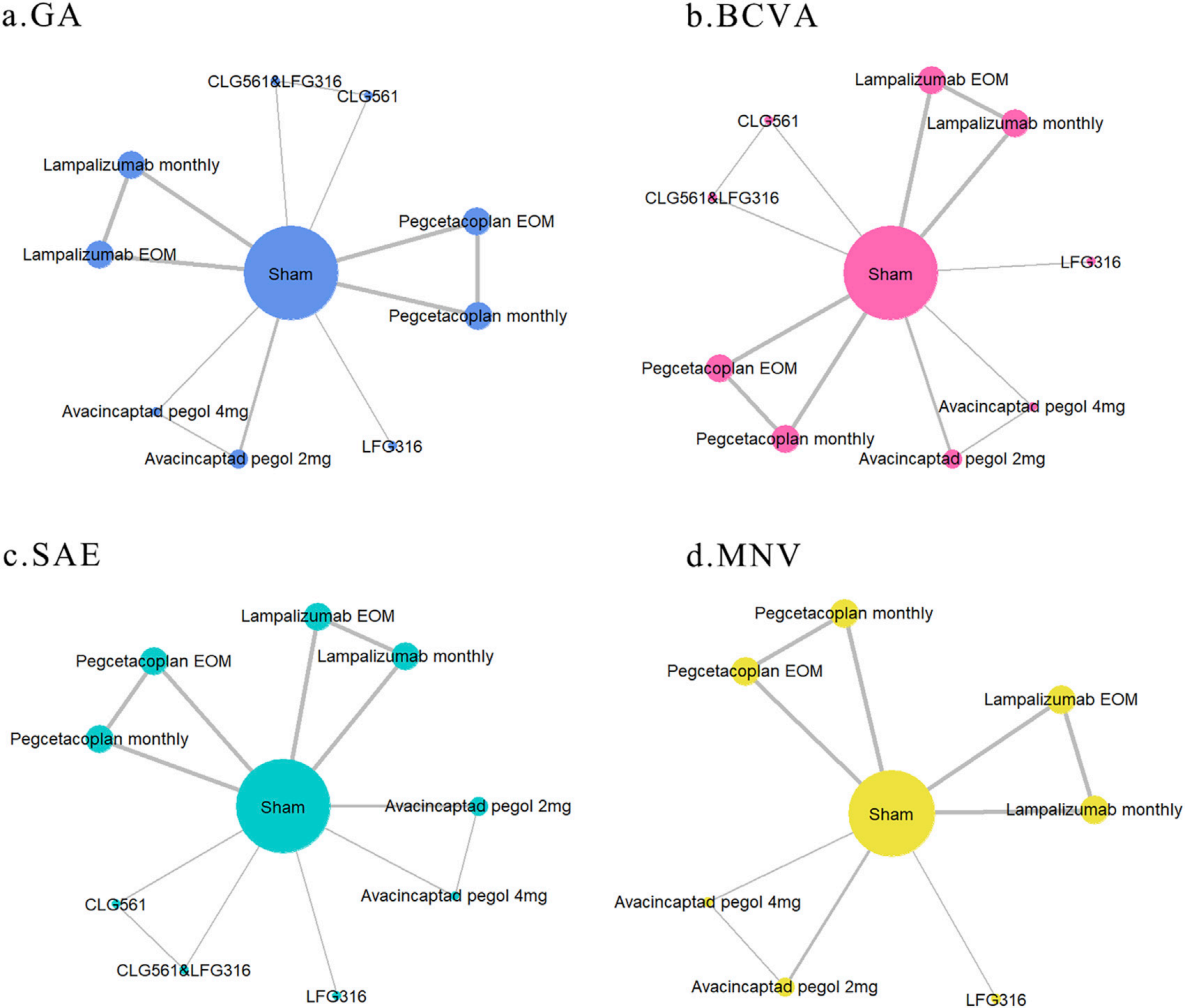
Network graph of different outcomes: **(A)** change in GA lesion size from baseline to month 12; **(B)** mean change in BCVA from baseline to month 12; **(C)** serious adverse events; and **(D)** macular neovascularization (MNV) in both eyes. The sizes of nodes and edges indicate the number of patients receiving the treatment and the number of studies for the comparison, respectively. The diameter of each node correlates positively with the number of patients included, while the thickness of the connecting lines reflects the number of direct comparisons. If nodes can form a closed loop, it indicates that these studies are capable of participating in simultaneous comparisons.

The network relationships between different outcomes in this study are illustrated in Figure 2 (network plot). All included RCTs that assessed changes in GA lesion size, BCVA, and SAE involved five drugs, nine interventions, and a total of 4,405 patients. This analysis resulted in 13 direct comparisons and four closed loops, as depicted in Figures 2A–C. Among these, the largest number of studies compared lampalizumab and pegcetacoplan with the sham (three studies). Additionally, there were two trials comparing changes in GA lesion size between avacincaptad pegol and sham groups, and one trial each involving CLG561 and LFG316. Excluding NCT02515942, nine registered RCTs reported on MNV. These studies included 4,292 patients and four drugs, resulting in 10 direct comparisons and three closed loops, as shown in Figure 2D. The intervention characteristic graph for outcomes is provided in Supplementary eAppendix S4, S1.

### 4.1 Assessment of model fit and inconsistency

All models were specified with a burn-in of 1,000 iterations, followed by 5,000 iterations with 20,000 adaptations. The Gelman–Rubin–Brooks plot showed whether there are outliers and the choice of the best effect model (see Supplementary eAppendix S5, S1). The posterior mean deviation between the consistency model and the inconsistency model is presented in Supplementary eAppendix S6, S1. The deviation report indicated acceptable consistency between the direct and indirect comparison results. Effect sizes of all outcomes were analyzed using a random-effects meta-analysis. Paired direct comparison meta-analysis with I^2^ statistic is shown in Supplementary eAppendix S7, S1.

The convergence diagnosis map, density map, and PSRF were used to evaluate the convergence and stability of the model. Each MCMC chain achieved stable convergence from the initial phase, with most of the chain fluctuation ranges covered by the overlapping area. Fluctuations of individual chains were not visually noticeable. The degree of convergence was satisfactory. The density map shows a smooth curve with a normal distribution and good stability (see Supplementary eAppendix S8, S1). The PSRF was below 1.05, indicating that the simulations performed were valid.

### 4.2 GA lesion size change at 1 year (mm^2^)

**FIGURE 3:**
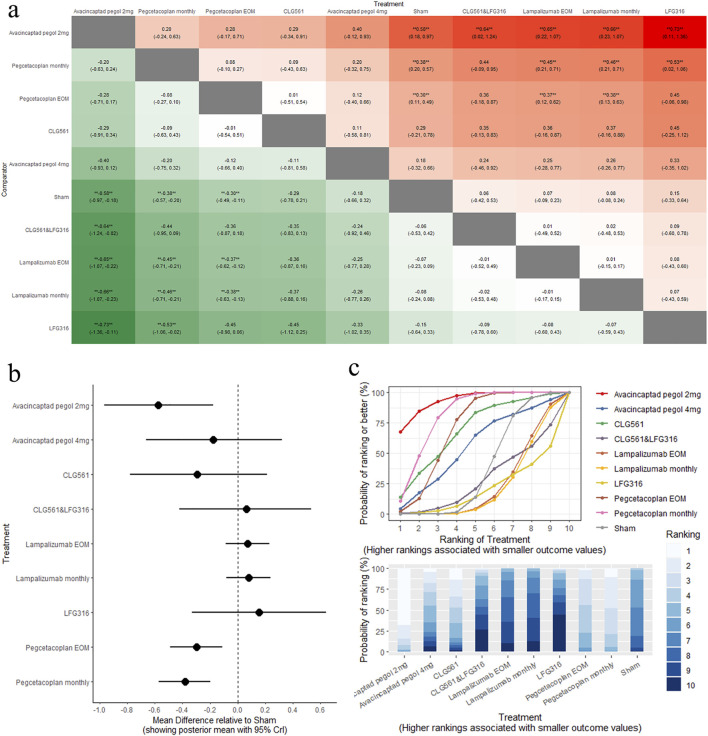
Comparison of GA change between treatments: **(A)** league tables: this chart presents the relative effectiveness of all intervention pairs along with their 95% credible intervals (CrI), enabling comparison between any two treatments. The symbol (**) indicates a statistically significant difference at the 95% CrI level between the “Treatment” and its “Comparator.” **(B)** forest plot: this plot displays the comparison results for each intervention against sham. The dashed line indicates the line of no effect, and the horizontal lines represent confidence intervals. If the confidence interval crosses the dashed line, the intervention shows no effect. **(C)** SUCRA and rankogram plots: presented as a line graph and bar chart, these plots show the ranking probabilities for each intervention.

The results of the pairwise comparisons of the GA lesion size are shown in Figure 3A (league tables). Direct comparison with sham revealed that avacincaptad pegol 2 mg (MD: −0.58, 95% CrI: −0.97 to −0.18), pegcetacoplan monthly (MD: −0.38, 95% CrI: −0.57 to −0.20), and pegcetacoplan every other month (EOM) (MD: −0.30, 95% CrI: −0.49 to −0.11) all led to significant reductions in GA lesion size. A comparison of the eight complement inhibitors and sham is shown in Figure 3B (forest plot). Indirect comparison between different interventions indicated that avacincaptad pegol 2 mg is superior to CLG561 & LFG316 (MD: −0.64, 95% CrI: −1.24 to −0.02), lampalizumab EOM (MD: −0.65, 95% CrI: −1.07 to −0.22), lampalizumab monthly (MD: −0.66, 95% CrI: −1.07 to −0.23), and LFG316 (MD: −0.73, 95% CrI: −1.36 to −0.11) in terms of GA lesion size change (Figure 3A). No statistically significant differences were found between other complement inhibitors. SUCRA analysis indicated that avacincaptad pegol 2 mg was the highest ranked for GA lesion size change (SUCRA = 93.55), followed by pegcetacoplan monthly (SUCRA = 81.37), with pegcetacoplan EOM being the lowest-ranked treatment option (SUCRA = 70.16), as shown in Figure 3C (SUCRA plot).

### 4.3 BCVA change at 1 year

**FIGURE 4:**
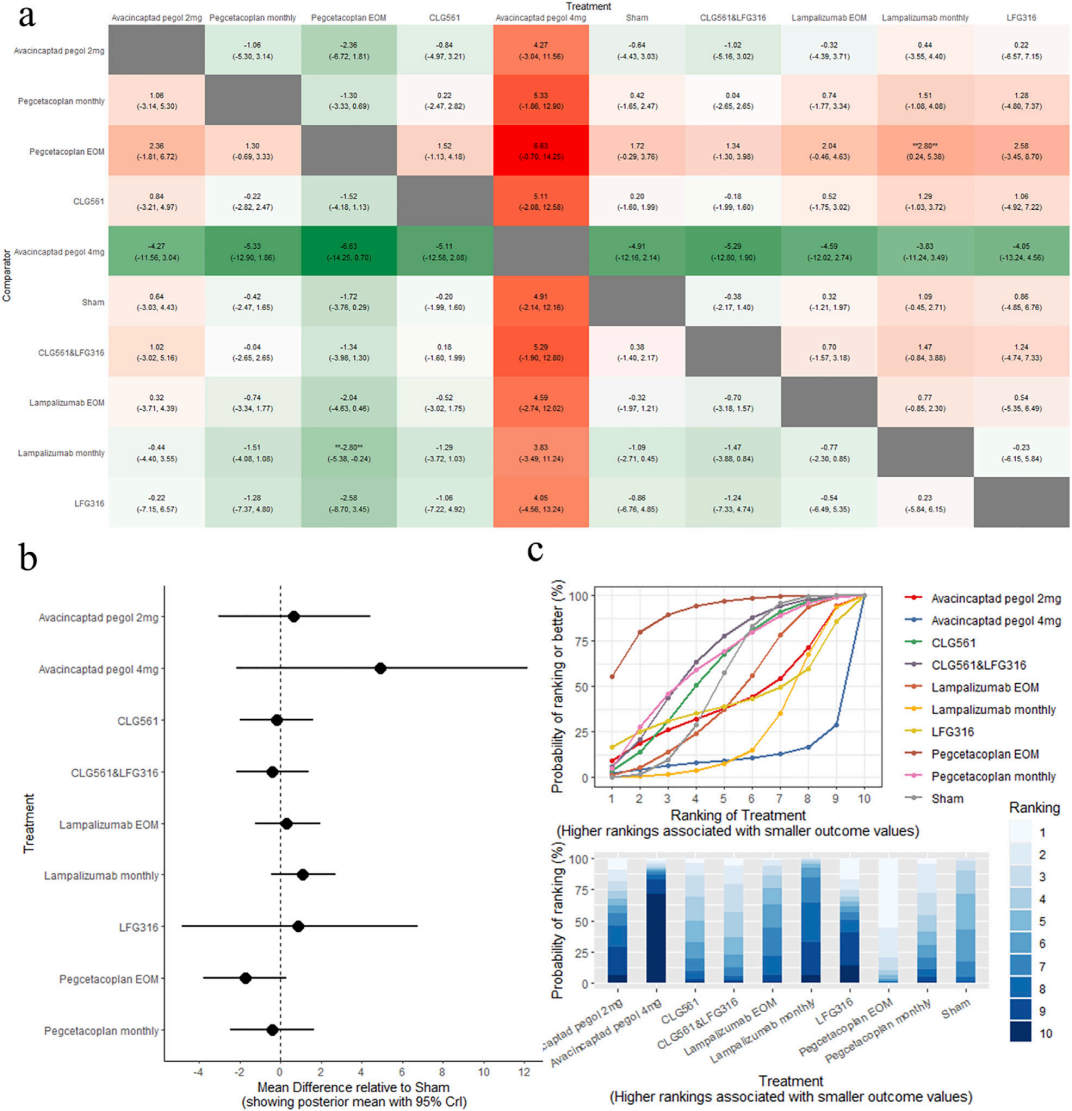
Comparison of BCVA change between treatments: **(A)** league tables: this chart presents the relative effectiveness of all intervention pairs along with their 95% credible intervals (CrI), enabling comparison between any two treatments. The symbol (**) indicates a statistically significant difference at the 95% CrI level between the “Treatment” and its “Comparator.” **(B)** forest plot: this plot displays the comparison results for each intervention against sham. The dashed line indicates the line of no effect, and the horizontal lines represent confidence intervals. If the confidence interval crosses the dashed line, the intervention shows no effect. **(C)** SUCRA and rankogram plots: presented as a line graph and bar chart, these plots show the ranking probabilities for each intervention.

The results of the pairwise comparisons of BCVA are shown in Figure 4A (league tables). No treatments demonstrated significant changes in BCVA compared to sham (Figure 4B, forest plot). Indirect comparison between different interventions indicated that pegcetacoplan EOM was superior to lampalizumab monthly (MD: −2.80, 95% CrI: −5.38 to −0.24). Pegcetacoplan EOM was the highest-ranked treatment option for BCVA (SUCRA = 90.49), followed by CLG561&LFG316(SUCRA = 65.75), and pegcetacoplan monthly (SUCRA = 63.43), as shown in Figure 4C (SUCRA plot).

### 4.4 SAE

Direct and indirect comparisons of all interventions did not show statistical differences in SAE (*P* > 0.05). The results of the pairwise comparisons of SAE are detailed in Supplementary eAppendix S9, S1 (including league tables and forest plot). Among the treatments, avacincaptad pegol 2 mg was ranked highest for SAE (SUCRA = 82.31), followed by CLG561&LFG316 (SUCRA = 74.04), and CLG561 (SUCRA = 68.64), as shown in Supplementary eAppendix S9C (SUCRA plot).

### 4.5 MNV

**FIGURE 5:**
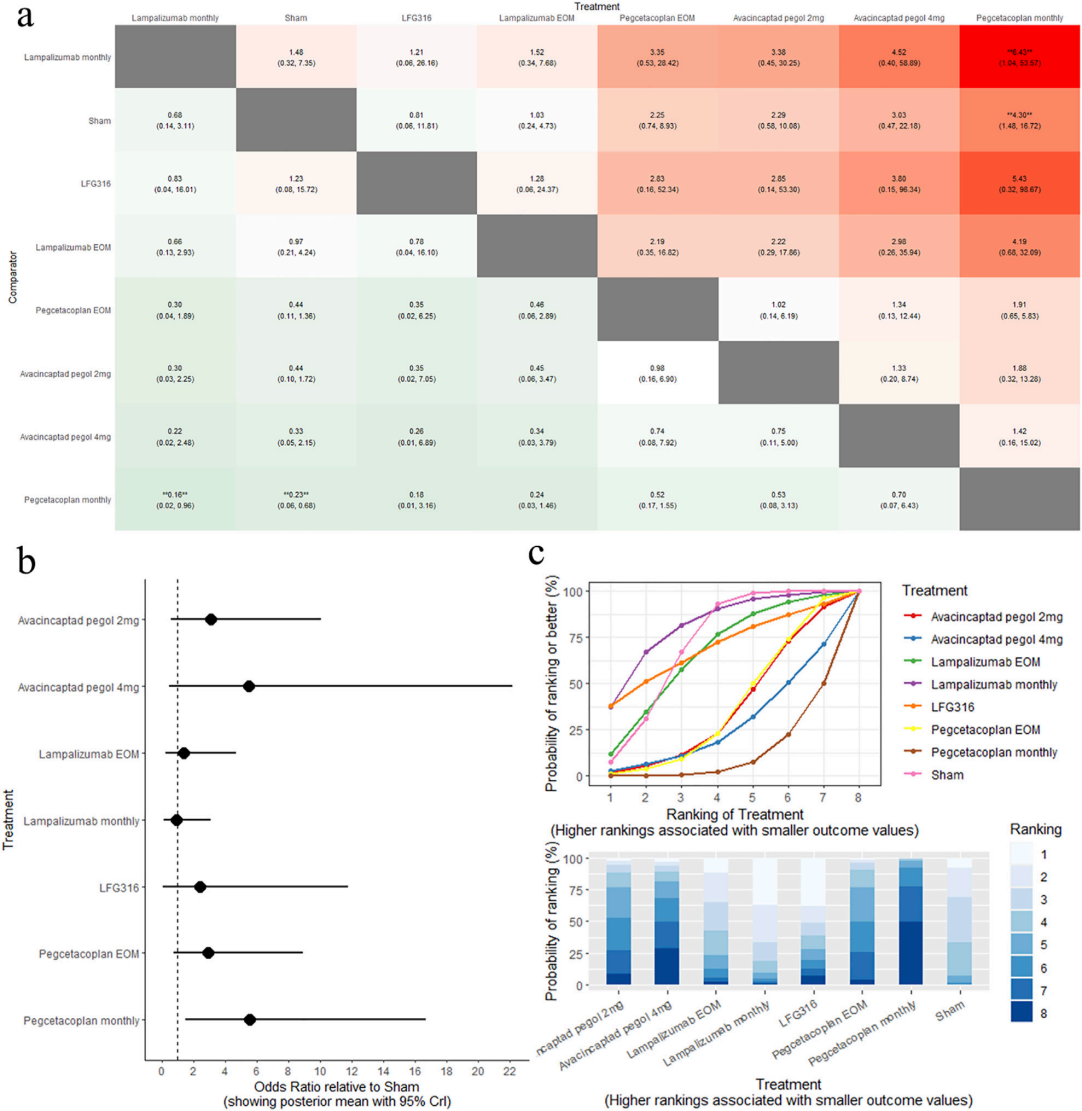
Comparison of MNV change between treatments: **(A)** league tables: this chart presents the relative effectiveness of all intervention pairs along with their 95% credible intervals (CrI), enabling comparison between any two treatments. The symbol (**) indicates a statistically significant difference at the 95% CrI level between the “Treatment” and its “Comparator.” **(B)** forest plot: this plot displays the comparison results for each intervention against sham. The dashed line indicates the line of no effect, and the horizontal lines represent confidence intervals. If the confidence interval crosses the dashed line, the intervention shows no effect. **(C)** SUCRA and rankogram plots: presented as a line graph and bar chart, these plots show the ranking probabilities for each intervention.

The results of the pairwise comparisons of the MNV are shown in Figure 5A (League tables). Direct comparison with sham revealed that pegcetacoplan monthly was associated with a high risk of MNV (OR: 4.30, 95% CrI: 1.48–16.72). A comparison of the eight complement inhibitors with the sham is provided in Figure 5B (forest plot). SUCRA analysis indicated that lampalizumab monthly was the highest-ranked treatment option for MNV (SUCRA = 81.49), followed by sham (SUCRA = 71.05), and LFG316 (SUCRA = 69.16), as shown in Figure 5C (SUCRA plot).

## 5 Discussion

This Bayesian NMA is the first technique to quantitatively evaluate the comparative effectiveness and safety of different complement inhibitors for improving GA in patients with AMD. We included a total of ten RCTs with 4,405 participants, covering four types of complement inhibitors: C3, C5, Factor D, and properdin inhibitors. The NMA results revealed that, among the five different complement inhibitors (pegcetacoplan, avacincaptad pegol, lampalizumab, CLG561, and LFG316), pegcetacoplan and avacincaptad pegol demonstrated clinically significant effects in reducing GA progression. SUCRA analysis results indicated that avacincaptad pegol 2 mg had the highest efficacy in treating GA, followed by pegcetacoplan monthly and pegcetacoplan EOM. While pegcetacoplan monthly was associated with a high risk of MNV, avacincaptad pegol 2 mg showed favorable outcomes in terms of SAE and MNV.

The primary findings of this study indicate that, among the existing complement inhibitors, avacincaptad pegol may offer greater efficacy and safety in reducing progression of GA in patients with AMD. The complement system is a highly regulated protein network that can be activated in a cascaded manner and operates at the interface of innate and adaptive immunity (Reis et al., 2019; Whitmore et al., 2015). It comprises three interconnected pathways: the classical pathway, alternative pathway, and lectin pathway. These pathways converge at the cleavage of complement C3 and C5, leading to the formation of the membrane attack complex, which is crucial for pathogen cell death (Anderson et al., 2010). Genetic studies have identified significant associations between AMD and variants of several complement-associated genes, including complement factor H, complement factor B, and complement component 3, indicating local complement protein expression within the eye (Riley-Gillis et al., 2023; Anderson et al., 2010; Kim et al., 2022). The complement system plays a key role in the pathogenesis of GA (Boyer et al., 2017; Zheng et al., 2022). Macular drusen, characteristic of early and intermediate stages of AMD, can progress to advanced AMD, which is associated with substantial vision loss. Some complement components, such as fragments of C3 and C5, have been found within drusen (Mullins et al., 2000; Schramm et al., 2014). GA represents the advanced stage of AMD, involving atrophy of the photoreceptors, RPE, and choriocapillaris. On average, patients exhibit an annual absolute GA progression rate of 1.59 mm^2^/y (95% CI, 1.46–1.71), corresponding to a square root-transformed GA progression rate of 0.26 mm/y (95% CI, 0.24–0.27) (Pfau et al., 2020).

Previous reviews have highlighted the therapeutic potential of C3 and C5 inhibitors in treating GA (Kim et al., 2022; Kim et al., 2021; Mastellos et al., 2023; Rathi et al., 2023). Pegcetacoplan, targeting C3 and C3b, was the first complement inhibitor to receive FDA approval for marketing (Heier et al., 2023). In our study, the paired meta-analysis results of pegcetacoplan EOM *versus* sham indicated an MD of −0.29 [95CrI%: −0.44 to −0.13]. Similarly, the results for pegcetacoplan administered monthly *versus* sham indicated an MD of −0.38 [95CrI%: −0.57 to −0.19]. These findings are consistent with those of research conducted by Tzoumas et al. (2023). Subsequently, avacincaptad pegol, targeting C5, also received marketing approval following favorable Phase III results. Our paired meta-analysis indicated that avacincaptad pegol 2 mg *versus* sham resulted in an MD of −0.58 [95CrI%: −0.96 to −0.20] (see Supplementary eAppendix S7, S1). Although no statistical difference were found in the indirect comparisons of these three interventions, the SUSAR ranking indicated that avacincaptad pegol 2 mg as the most effective, followed by pegcetacoplan monthly and pegcetacoplan EOM. This suggests that avacincaptad pegol may be more potential than pegcetacoplan in improving outcomes related to GA progression. Furthermore, indirect comparison between avacincaptad pegol and pegcetacoplan and other therapies also reported significant differences in efficacy. Research has indicated that intravitreal injections with higher volume may lead to increased intraocular pressure (IOP) (Meyer et al., 2023; El Chehab et al., 2016). In our study, the rate of increased IOP increased with complement inhibitors compared to sham, with avacincaptad pegol 4 mg showing the highest rate of increased IOP at 22.8%.

None of the complement inhibitors demonstrated a statistically significant difference in improving BCVA compared to sham at 1 year, which is consistent with the findings of Anubhav Garg et al. (2023). Visual function is crucial to both patients and physicians, and BCVA is a fundamental measure visual function. However, it does not entirely capture meaningful functional visual decline throughout the macula and does not show a good correlation with patient’s symptoms (Sadda et al., 2016). Important visual function results also include contrast sensitivity, color, depth, and motion, as well as field of view (Bennett et al., 2019). The FDA encourages the development of new clinical endpoints to measure clinically significant effects in patients with retinal diseases (US Department of Health and Human Services FaDA, 2020), especially for studies focusing on the recovery or improvement of vision in patients with severe visual impairment (Bailey and Lovie-Kitchin, 2013). Dolly S Chang‘s study found that perilesional or responding macular sensitivity measured by microperimetry was a more sensitive endpoint than mean macular sensitivity for detecting functional deterioration (Chang et al., 2024). Additionally, the Multi-Luminance Mobility Test is considered to evaluate an individual's functional vision and ability to perform visually independent daily activities, providing standardized and quantifiable assessments for clinical trials (Chung et al., 2018; Maguire et al., 2019; Girgis and Lee, 2023). Several studies have reported emerging endpoints such as low luminance visual acuity (Liao et al., 2020; Holz et al., 2018) and National Eye Institute Visual Functioning Questionnaire 25-item Version Composite Score (Heier et al., 2023; Holz et al., 2018). However, since most studies have not reported these, we did not consider them in this analysis.

Direct and indirect comparisons of all interventions did not reveal statistically significant differences in SAEs, which is consistent with findings from other studies on intravitreal drugs (Garg et al., 2023). Additionally, our study found that pegcetacoplan monthly was associated with a high risk of MNV (OR: 4.30, 95% CrI: 1.48–16.72). In addition to pegcetacoplan monthly, Tzoumas et al., (2023) also suggested that avacincaptad pegol might substantially increase the risk of MNV or exudative AMD compared to sham over a year. Despite the fact that there is much hope and marketing about tissue preservation with pegcetacoplan, there are many safety and efficacy concerns that may interrupt widespread adoption by clinicians (Schachar, 2023; Nadeem et al., 2023). In contrast, avacincaptad pegol 2 mg showed favorable outcomes in terms of SAEs and MNV. Overall, based on both efficacy and safety, avacincaptad pegol 2 mg appears to be the most promising choice among the complement inhibitors for treating GA secondary to AMD.

Although avacincaptad pegol and pegcetacoplan have received FDA approval for the treatment of GA, this approve is primarily based on their efficacy in slowing GA progression, as measured by anatomical endpoints. Phase 3 clinical trials, however, have not demonstrated any significant functional improvement after 1 or 2 years of treatment (Khanani et al., 2023; Heier et al., 2023). Do these drugs represent “clinically relevant outcomes”? Given that visual loss typically manifests only in the advanced stages of GA, utilizing visual acuity as the primary outcome measure in GA trials would necessitate study durations extending well beyond those of most large-scale randomized clinical trials. This makes it challenging to identify practical and clinically meaningful endpoints for GA. While FAF imaging serves as the standard for FDA assessment of increase in GA size, the correlation between functional changes and FAF at the GA boundary remains unclear. Consequently, there is a notable disconnect between anatomical and functional outcomes in existing clinical trials. Csaky et al., (2024) suggested that future clinical trials should combine FAF and optical coherence tomography imaging, while quantifying GA boundaries and incorporating functional endpoints, in order to develop more effective treatment strategies.

## 6 Limitation

This research has several limitations. First, the analysis is based on a limited number of studies comparing sham *versus* five complement inhibitors. The included studies differed in patient populations, design, and statistical methods, contributing to potential heterogeneity that may affect the precision of our estimates. Factors such as patient age and lesion location could further contribute to this heterogeneity. Additionally, the relatively small number of randomized controlled trials (RCTs) may impact the robustness of some comparisons. Second, using a 1-year endpoint may underestimate the long-term benefits or risks associated with complement inhibitors. Third, the lack of statistically significant differences in changes in BCVA suggests that the chosen outcome measure might lack sensitivity. Given the multifactorial nature of visual function, a more nuanced evaluation that includes patient-reported outcomes and functional vision assessments is needed to fully capture the impact of complement inhibitors on visual acuity. Despite these limitations, significant progress is being made in the development and research of complement inhibitors for treating GA. More large-scale real-world studies are necessary to confirm these findings in the future.

## 7 Conclusion

The results of this network meta-analysis indicate that avacincaptad pegol and pegcetacoplan have clinically significant effects in reducing the progression of GA in patients with AMD. The Bayesian NMA indicates that avacincaptad pegol 2 mg is the most effective complement inhibitor with better safety for improving GA in AMD.

## Data Availability

The original contributions presented in the study are included in the article/Supplementary Material, further inquiries can be directed to the corresponding authors.

## Abbreviations

AMD, age-related macular degeneration; BCVA, best corrected visual acuity; BUGSnet, Bayesian inference using Gibbs sampling to conduct network; C, complement factor; CNV, choroidal neovascularization; CI, confidence interval; CrI, credible interval; DIC, deviance information criterion; EOM, every other month; ETDRS, Early Treatment Diabetic Retinopathy Study; FAF, fundus autofluorescence; FDA, Food and Drug Administration; GA, geographic atrophy; IOP, intraocular pressure; MCMC, Markov Chain Monte Carlo; MD, mean difference; MNV, macular neovascularization; NMA, network meta-analysis; OR, odds ratio; RCT, randomized controlled trials; RPE, retinal pigment epithelium; PRISMA, Preferred Reporting Items for Systematic reviews and Meta-Analysis; PSRF, potential scale reduction factor; SAE, serious adverse event; SUCRA, Surface Under the Cumulative Ranking Curve; WHO, World Health Organization.
