# Supplementary material for: Efficacy and safety of complement inhibitors in patients with geographic atrophy associated with age-related macular degeneration: a network meta-analysis of randomized controlled trials
Source: Front Pharmacol. 2024 Nov 12;15:1410172. doi: 10.3389/fphar.2024.1410172 (PMC11589381; doi:10.3389/fphar.2024.1410172)

**Supplement material**

**Appendix 1: Search Criteria**

**Cochrane Central search strategy**

#1=MeSH descriptor: [Geographic Atrophy] explode all trees

#2= (Atrophies, Geographic or Atrophy, Geographic or Geographic Atrophies or Dry Macular Degeneration or Degeneration, Dry Macular or Degenerations, Dry Macular or Dry Macular Degenerations or dry age-related macular degeneration or Macular Degeneration, Dry or Macular Degenerations, Dry or Non-exudative Age-Related Macular Degeneration):ti,ab,kw (Word variations have been searched)

#3=#1 or #2

#4=MeSH descriptor: [Complement Inactivating Agents] explode all trees

#5= (Agents, Complement Inactivating or Inactivating Agents, Complement or Complement Inhibitors or Inhibitors, Complement or Complement Inhibiting Agents or Agents, Complement Inhibiting or Inhibiting Agents, Complement or Complement Inhibitor or Inhibitor, Complement or Complement Cytolysis Inhibiting Agents or Complement System Proteins): ti,ab,kw (Word variations have been searched)

#6=#4 or #5

#7=#3 and #6

**Web of Science** **Core Collection search strategy**

1: TS= (Macular Degeneration OR AMD OR nAMD OR ARMD OR Geographic Atrophy OR Dry Macular Degeneration OR Non-exudative Age-Related Macular Degeneration)

2: TS= (Complement OR C3 OR C5 OR Eculizumab OR solaris OR Lampalizumab OR avancincaptad pegol OR ARC‐1905 OR zimbra OR tesiclolumab OR lg316 computation OR POT 4 OR pots OR cl1561 OR APL‐2 OR ixtayopan OR Complement System Proteins)

3: #2 AND #1

**PubMed search strategy**

**(((((((randomized controlled trial[Publication Type]) OR (randomized[Title/Abstract] OR randomised[Title/Abstract])) OR (placebo[Title/Abstract])) OR (randomly[Title/Abstract])) OR (trial[Title/Abstract])) OR (groups[Title/Abstract])) NOT (("exp"[All Fields] AND "animals"[All Fields]) NOT ("exp"[All Fields] AND "animals"[All Fields] AND ("exp"[All Fields] AND "animals"[All Fields])))) AND ((((((((((((exp macular degeneration) OR (retinal degeneration)) OR (retinal neovascularization)) OR (choroidal neovascularization)) OR (exp macula lutea)) OR (maculopathy$[Text Word])) OR (("macul"[Text Word] OR "retina"[Text Word] OR "choroid"[Text Word]) AND "degener"[Text Word])) OR (("macul"[Text Word] OR "retina"[Text Word] OR "choroid"[Text Word]) AND "degener"[Text Word])) OR (macula[Text Word] AND lutea[Text Word])) OR (AMD[Text Word] OR ARMD[Text Word] OR CNV[Text Word])) OR (geographic[Text Word] AND atrophy[Text Word])) AND ((((((((((exp complement system proteins) OR (complement[Text Word] AND (cascad[Text Word] OR inhibit[Text Word] OR pathway[Text Word]))) OR (C3[Text Word] OR C5[Text Word])) OR (eculizumab[Text Word] OR soliris[Text Word])) OR (lampalizumab[Text Word])) OR (compstatin[Text Word])) OR (POT 4[Text Word] OR POT4[Text Word])) OR (Avacincaptad pegol[Text Word] OR ARC‐1905[Text Word] OR Zimura[Text Word])) OR (Tesidolumab[Text Word] OR LFG316[Text Word])) OR (CLG561[Text Word] OR APL‐2[Text Word] OR Fovista[Text Word] OR Pegpleranib[Text Word] OR Pegcetacoplan[Text Word] OR HMR 59[Text Word] OR HMR59[Text Word] OR Iptacopan[Text Word])))**

**LWW Medical Journals search strategy**

1.exp retina degeneration/

2.retina neovascularization/

3.subretinal neovascularization.mp. or subretinal neovascularization/

4.(AMD or ARMD or CNV).mp. [mp=title, abstract, heading word, drug trade name, original title, device manufacturer, drug manufacturer, device trade name, keyword heading word, floating subheading word, candidate term word]

5.(Dry Macular or Degenerations).mp. [mp=title, abstract, heading word, drug trade name, original title, device manufacturer, drug manufacturer, device trade name, keyword heading word, floating subheading word, candidate term word]

6.age related macular degeneration/dr, dt [Drug Resistance, Drug Therapy]

7.1 or 2 or 3 or 4 or 5 or 6

8.exp complement/

9.(complement adj3 (cascad$ or inhibit$ or pathway$)).tw.

10.(C3 or C5).tw.

11.(eculizumab or soliris).tw.

12.lampalizumab.tw.

13.compstatin.tw.

14.(POT 4 or POT4).tw.

15.(Avacincaptad pegol or Zimura).tw.

16.(Tesidolumab or LFG316).tw.

17.(Fovista or Pegpleranib or Pegcetacoplan or Iptacopan).tw.

18.8 or 9 or 10 or 11 or 12 or 13 or 14 or 15 or 16 or 17

19.APL-2.tw.

20.18 or 19

21.exp comparative study/

22.exp evaluation/

23.exp prospective study/

24.(control$ or prospectiv$ or volunteer$).tw.

25.21 or 22 or 23 or 24

26.7 and 20 and 25

**ClinicalTrials.gov search strategy**

(Macular Degeneration OR AMD OR nAMD OR ARMD OR Geographic Atrophy OR Dry Macular Degeneration OR Non-exudative Age-Related Macular Degeneration) AND (Complement OR C3 OR C5 OR Eculizumab OR Soliris OR Lampalizumab OR Avacincaptad pegol OR ARC‐1905 OR Zimura OR Tesidolumab OR LFG316 Compstatin OR POT 4 OR POT4 OR CLG561 OR APL‐2 OR Iptacopan OR Complement System Proteins)

**WHO ICTRP search strategy**

Condition = Macular Degeneration OR AMD OR nAMD OR ARMD OR Geographic Atrophy OR Dry Macular Degeneration OR Non-exudative Age-Related Macular Degeneration AND Intervention = Complement OR C3 OR C5 OR Eculizumab OR Soliris OR Lampalizumab OR Avacincaptad pegol OR ARC‐1905 OR Zimura OR Tesidolumab OR LFG316 Compstatin OR POT 4 OR POT4 OR CLG561 OR APL‐2 OR Iptacopan OR Complement System Proteins

**Appendix 2: Functions of the BUGSnet package**

Table 1. Functions of the BUGSnet package

| Purpose | Function | Description |
| --- | --- | --- |
| Data preprocessing | data.prep() | Prepare data for further processing |
| Network description | net.tab() | Descriptive statistics of evidence networks |
|  | net.plot() | Draw the evidence network |
| Homogeneity assessment | pma() | Heterogeneity statistics to obtain the results of Pairwise comparison |
|  | data.plot() | Draw characteristic graph of research or interventions |
| Consistency assessment | nma.compare() | Compare consistency and inconsistency models |
| Network meta-analysis | nma.model() | Specify NMA models |
|  | nma.run() | Run NMA |
| Model evaluation | nma.diag() | Obtain trajectory graphs and other convergence diagnostic values |
|  | nma.fit() | Obtaining lever graphs and deviation information standard (DIC) values |
| Result output | nma.forest() | Draw an NMA forest graphs |
|  | nma.league() | Draw NMA ranking table |
|  | nma.rank() | Obtain table and graphical results for treatment ranking |
|  | nma.regplot() | Estimated relative treatment effect graphs |

**Appendix 3:** **Risk of bias graph for each included study**


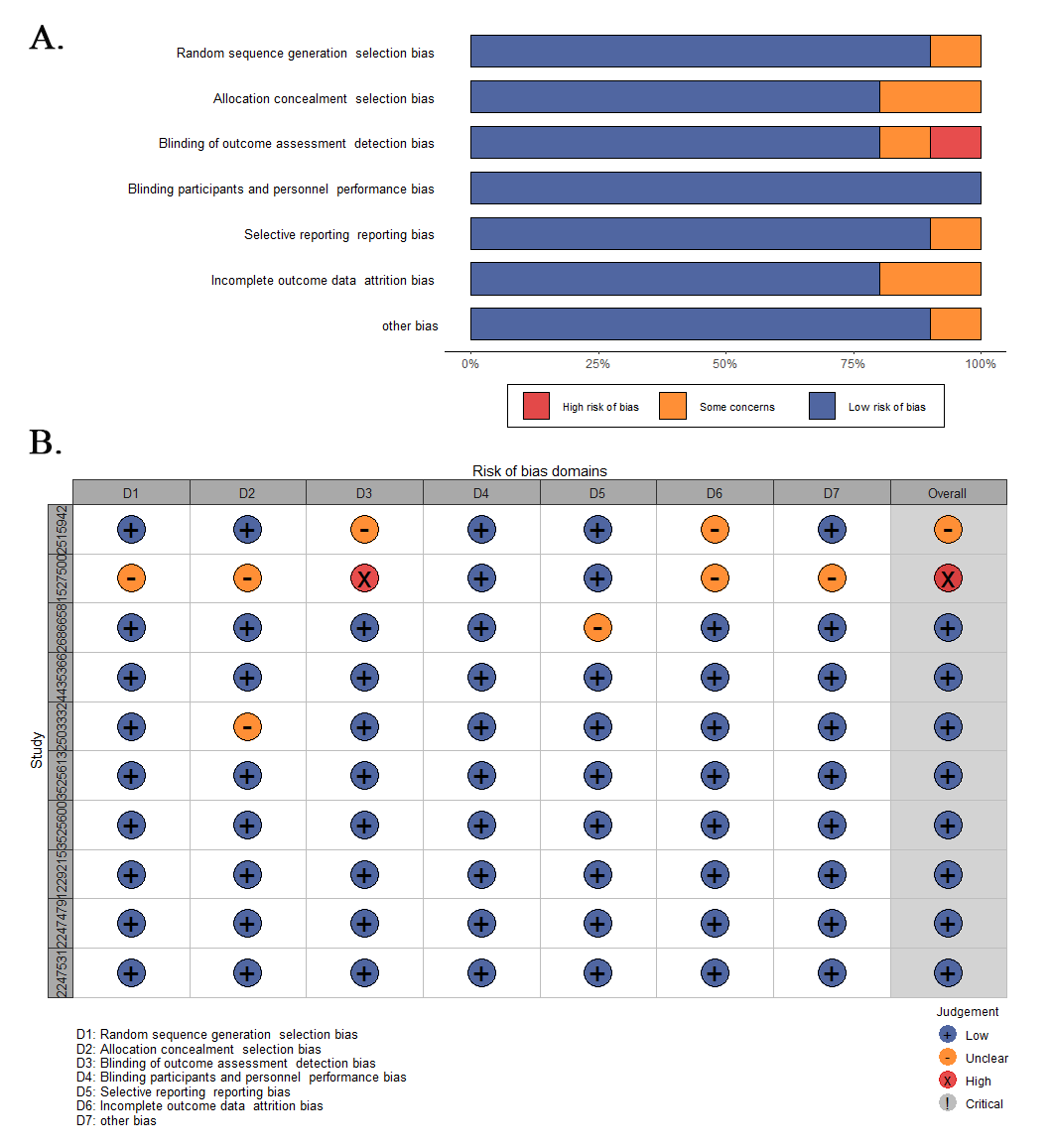


**Appendix 4:** **Interventions characteristic of outcomes**


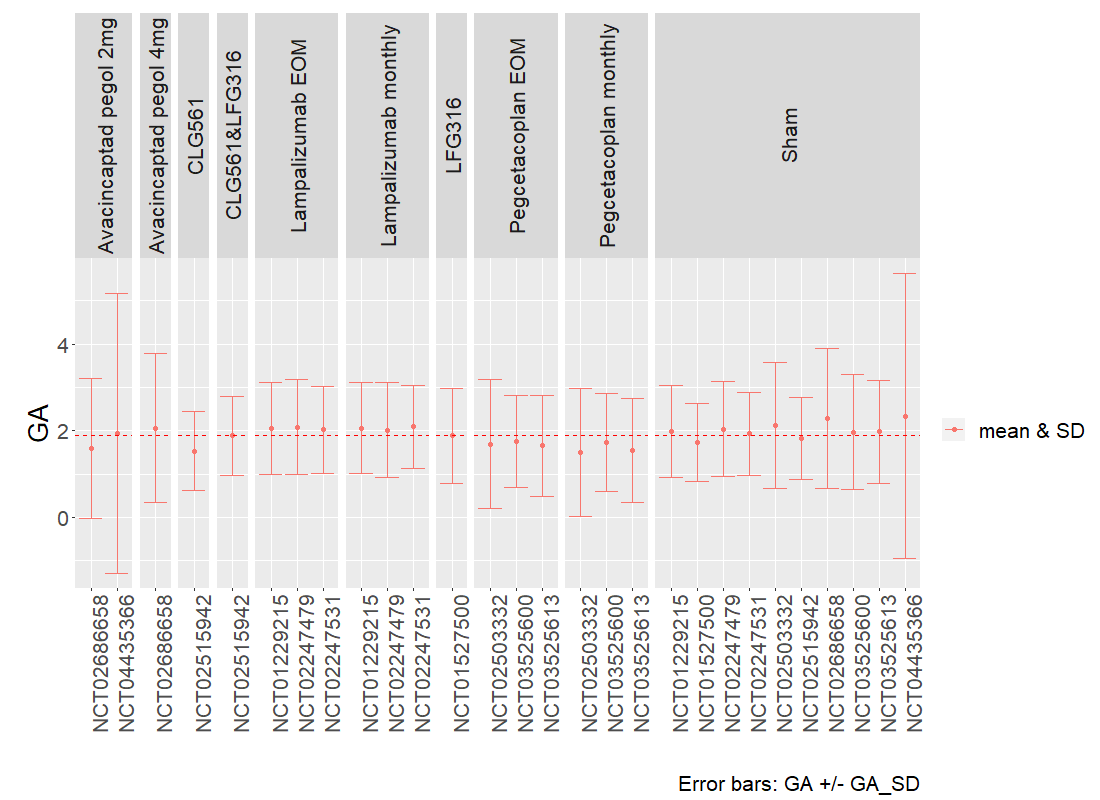


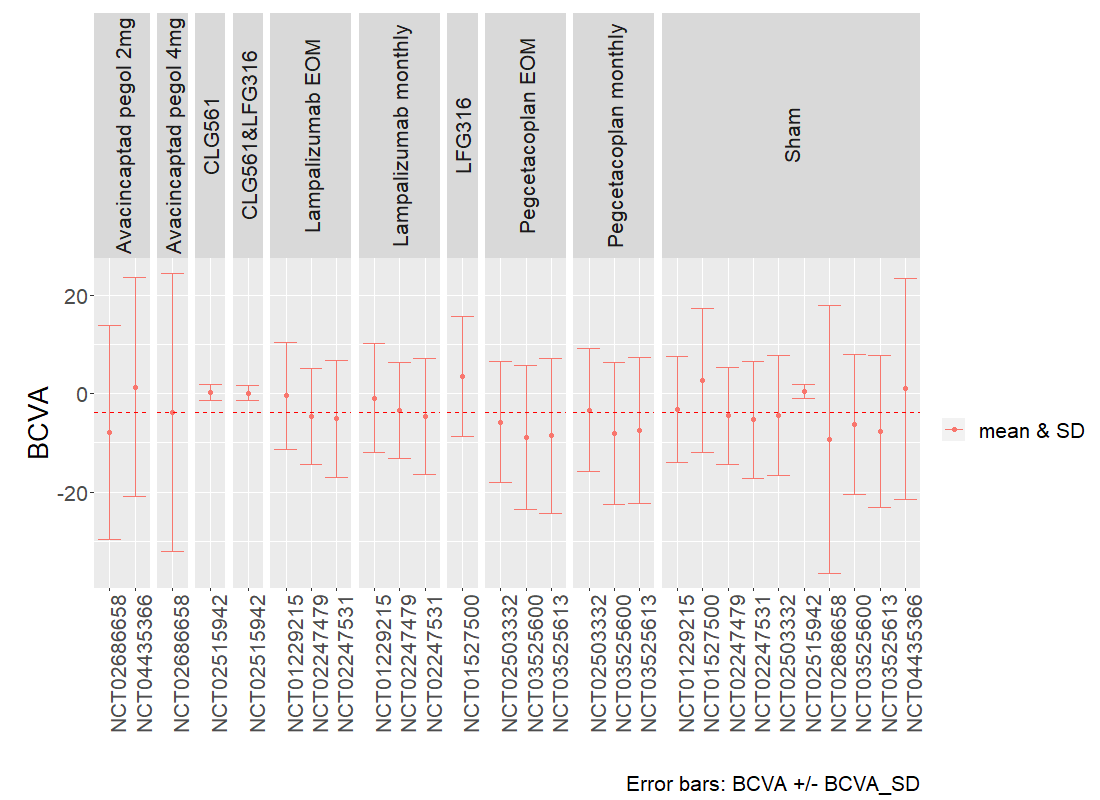

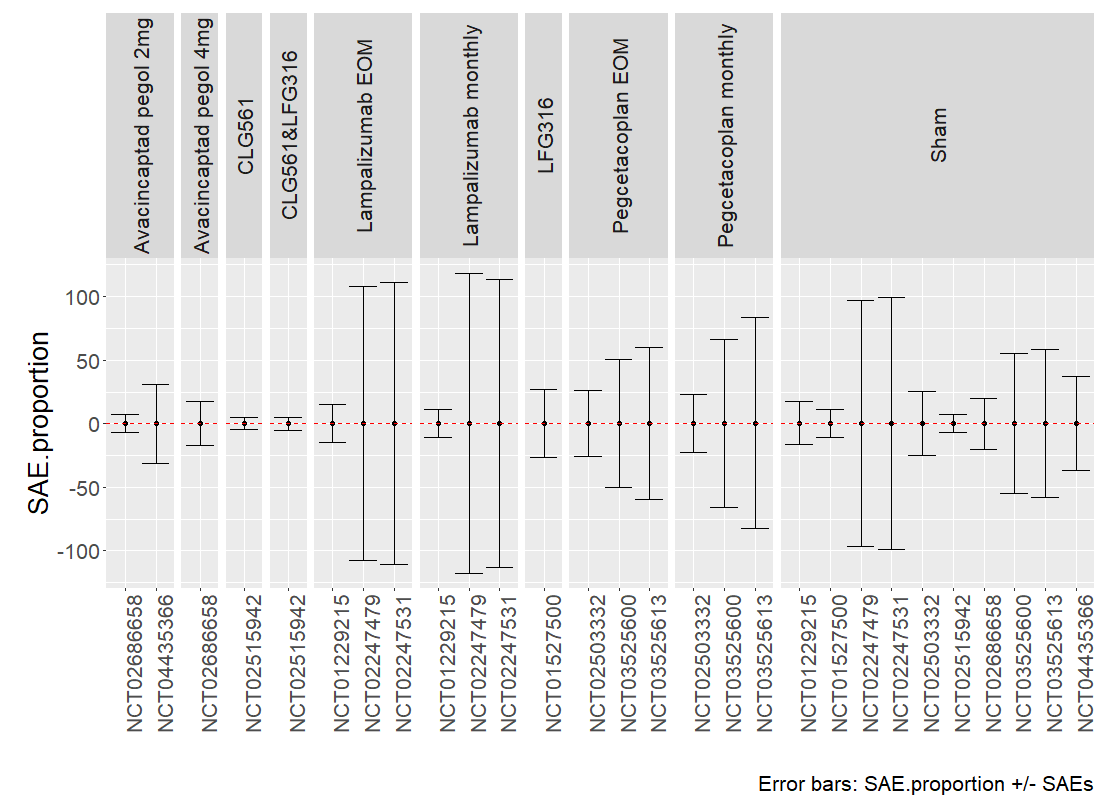

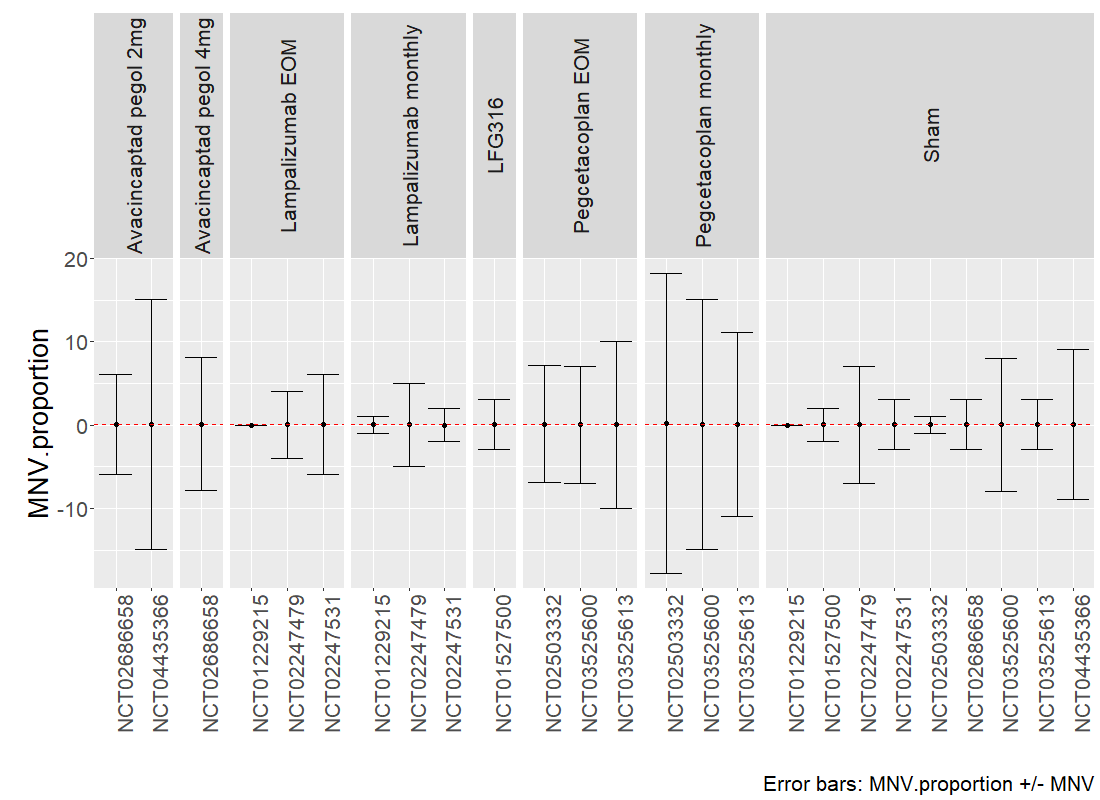


**Appendix 5:** **Gelman–Rubin–Brooks plot of outcomes**


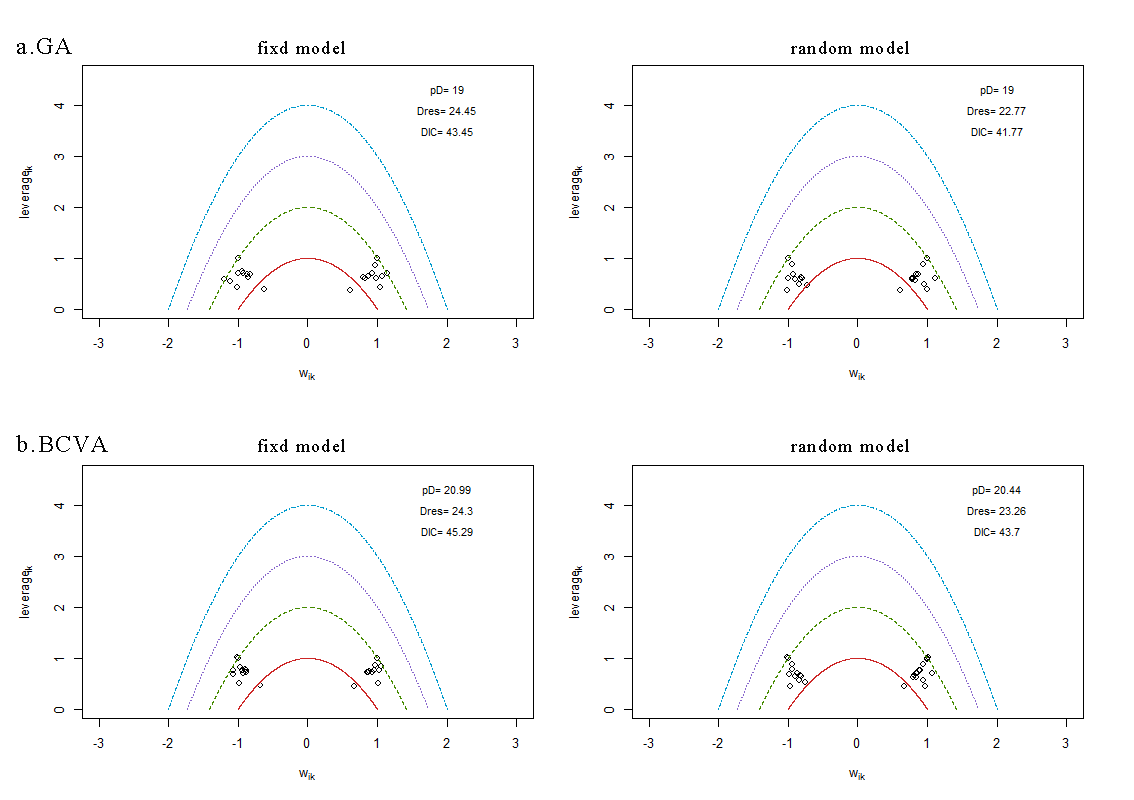


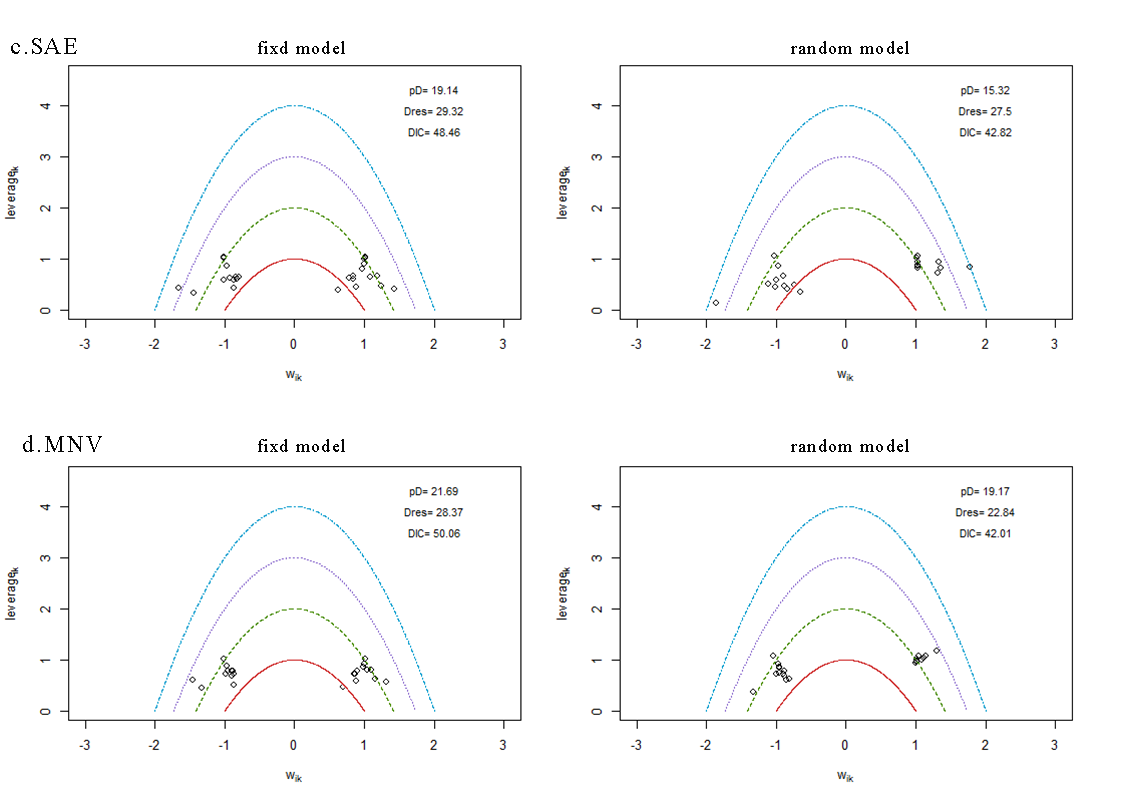


**Appendix 6:** **Consistency vs Inconsistency plot of outcomes**


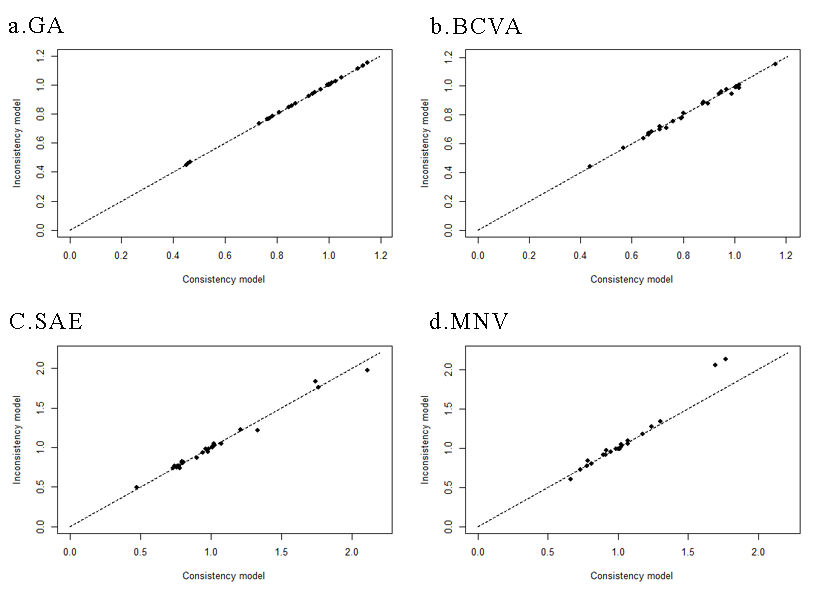


**Appendix 7:** **Paired direct comparison Meta-analysis**

**GA**

(1) Avacincaptad pegol 2mg VS sham


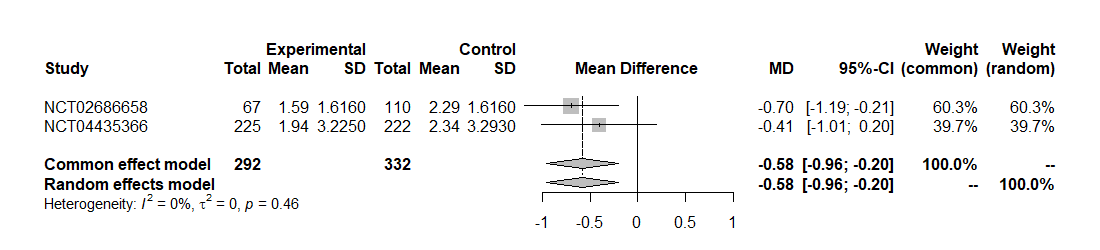


(2) Pegcetacoplan EOM VS sham


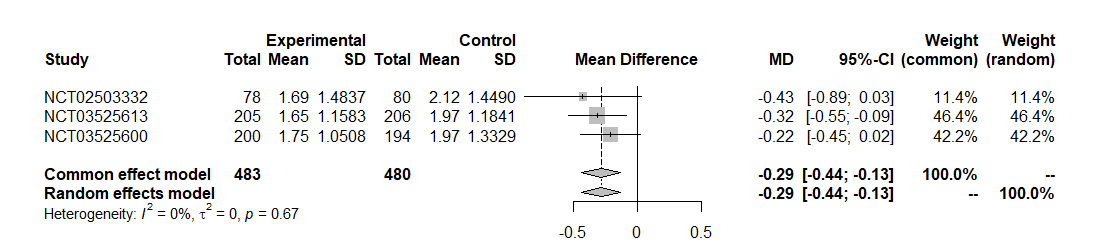


(3) Pegcetacoplan monthly VS sham


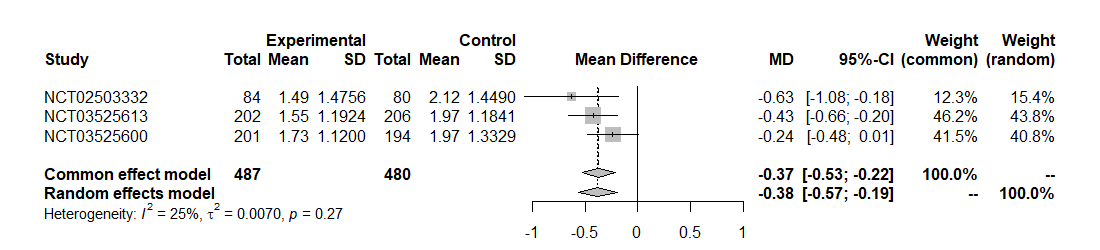


(4) Lampalizumab EOM VS sham


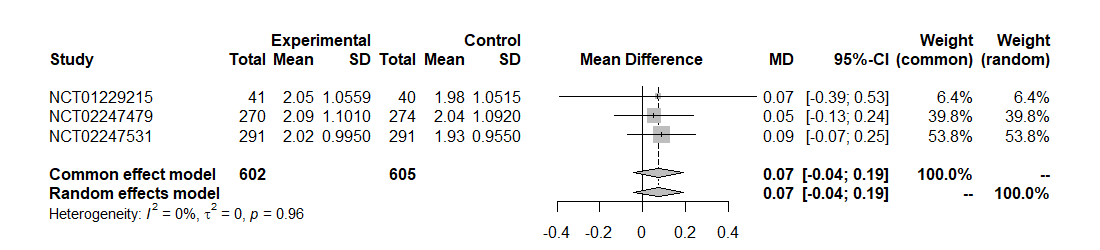


(5) Lampalizumab monthly VS sham


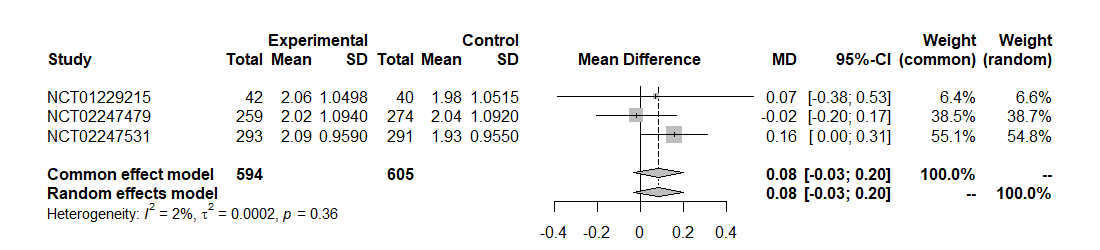


**BCVA**

(1) Avacincaptad pegol 2mg VS sham


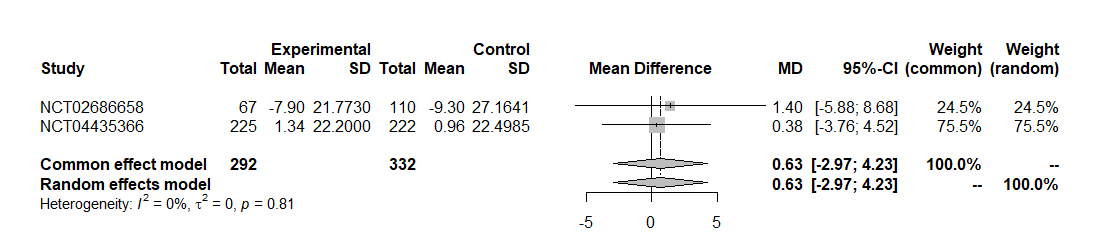


(2) Pegcetacoplan EOM VS sham


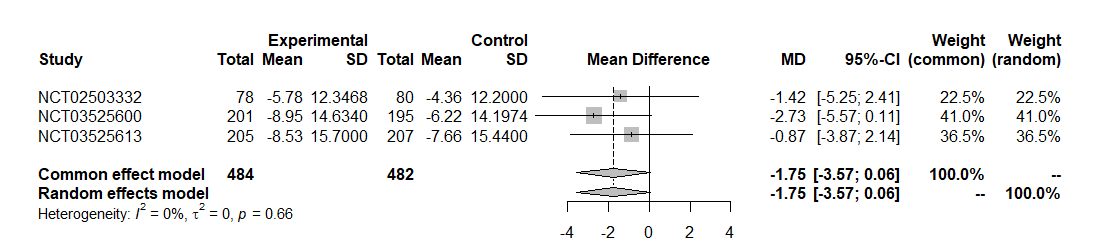


(3) Pegcetacoplan monthly VS sham


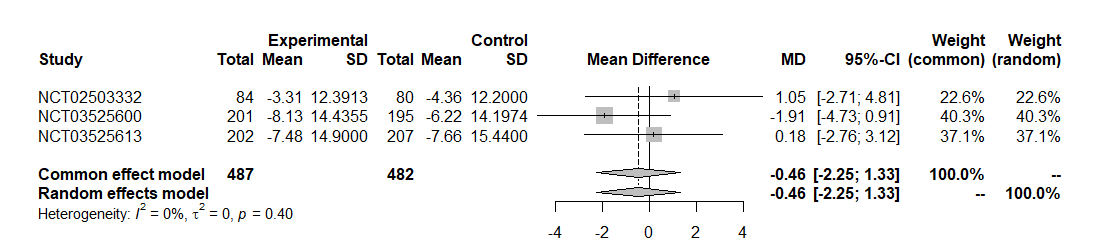


(4) Lampalizumab EOM VS sham


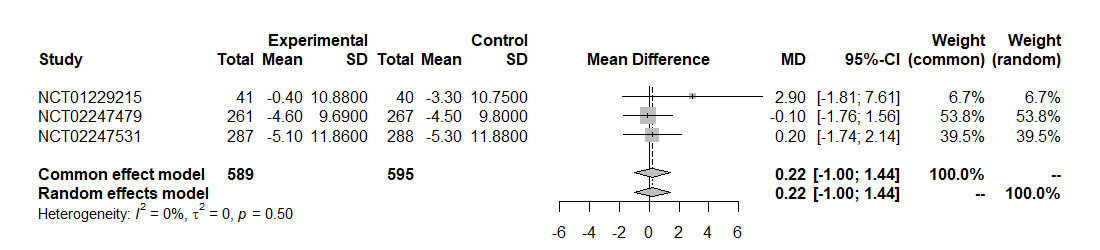


(5) Lampalizumab monthly VS sham


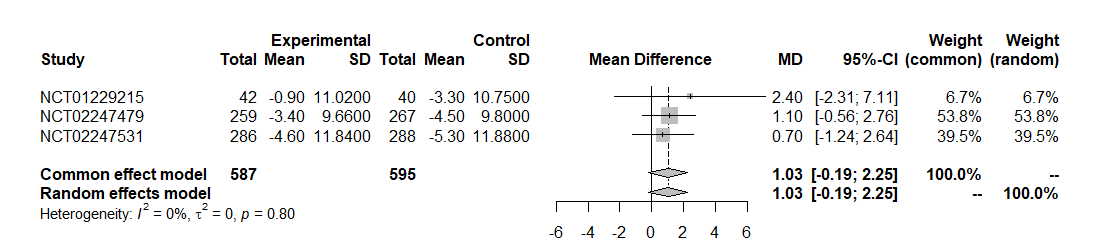


**SAE**

(1) Avacincaptad pegol 2mg VS sham


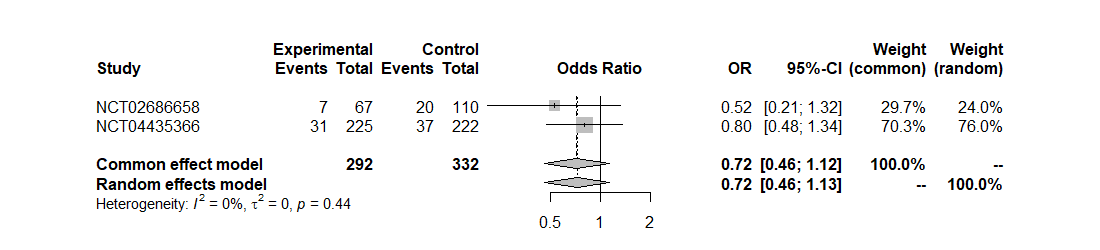


(2) Pegcetacoplan EOM VS sham


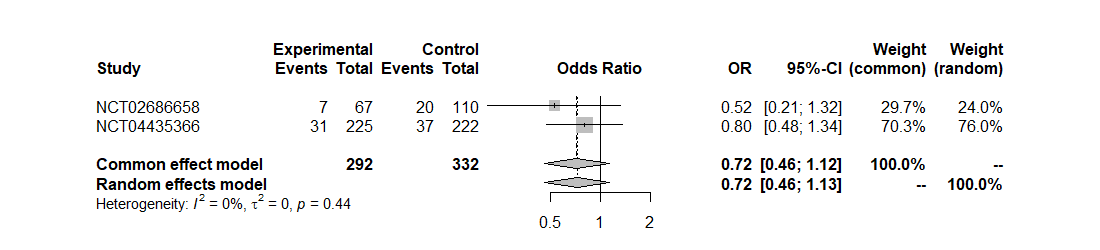


(3) Pegcetacoplan monthly VS sham


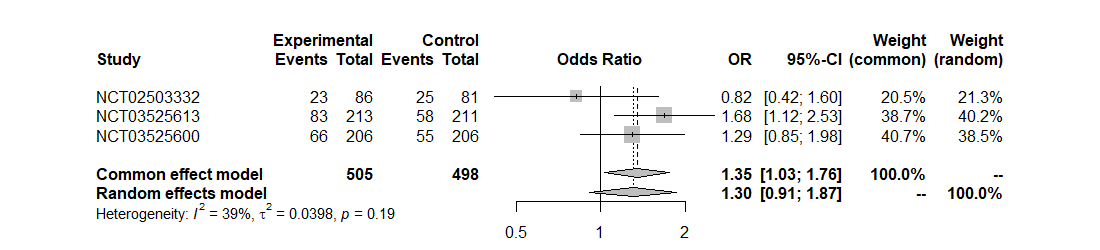


(4) Lampalizumab EOM VS sham


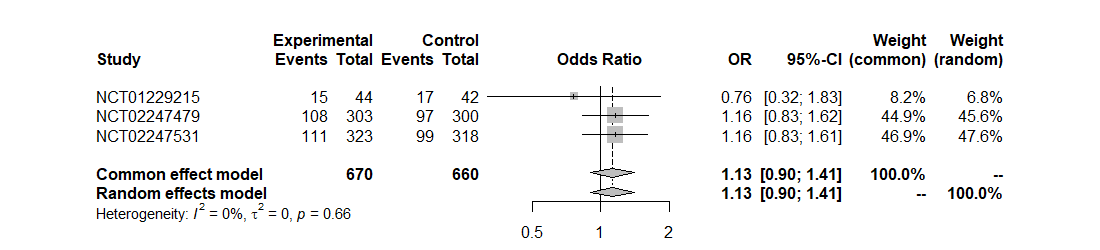


(5) Lampalizumab monthly VS sham


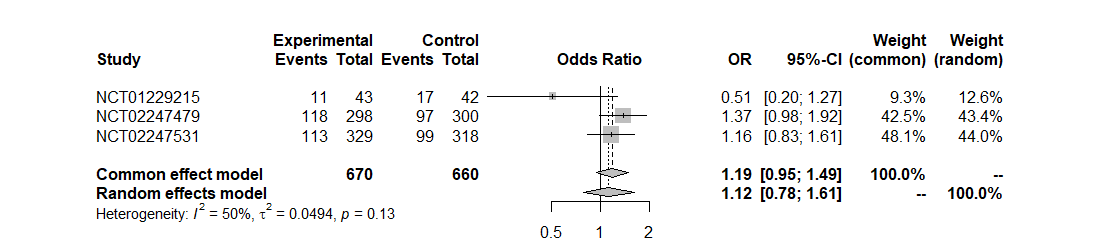


**MNV**

(1) Avacincaptad pegol 2mg VS sham


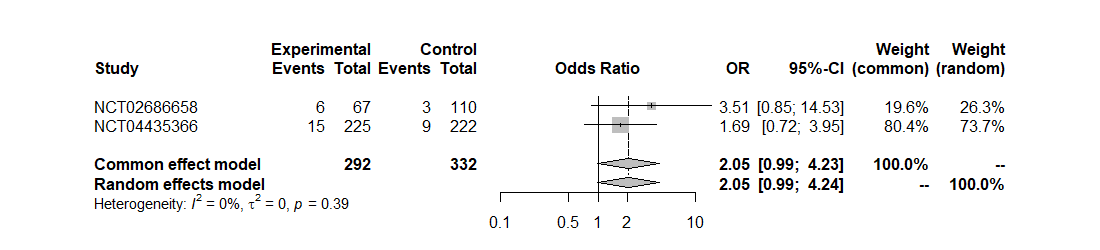


(2) Pegcetacoplan EOM VS sham


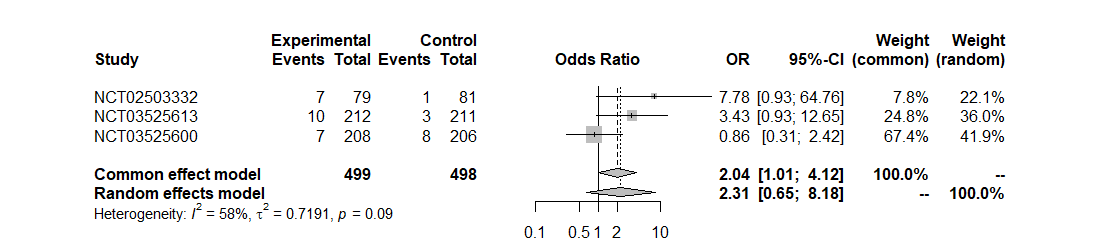


(3) Pegcetacoplan monthly VS sham


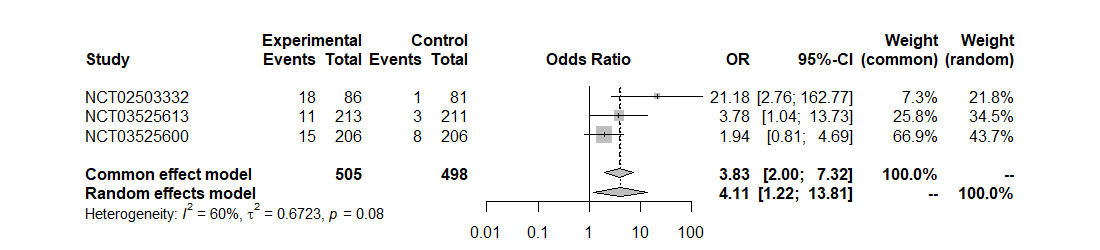


(4) Lampalizumab EOM VS sham


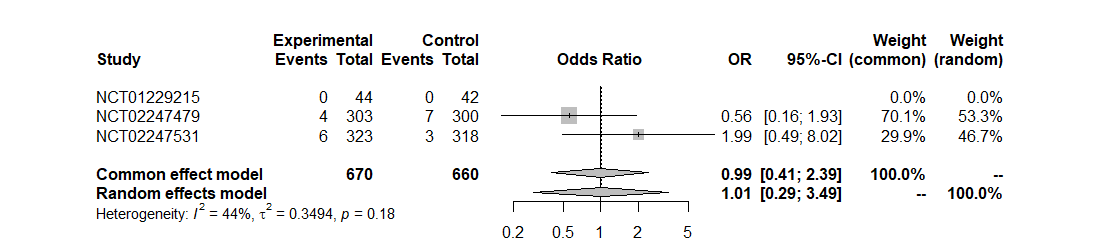


(5) Lampalizumab monthly VS sham


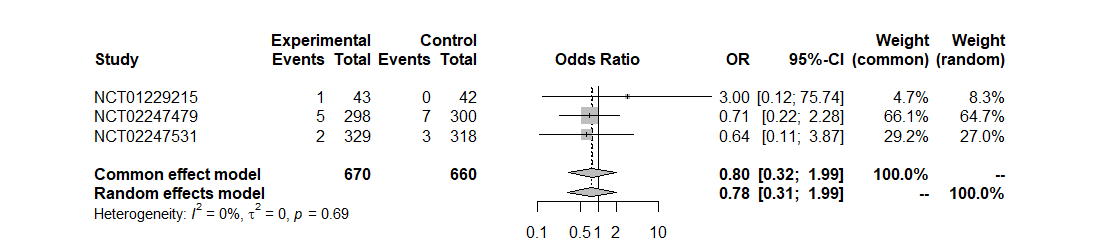


Appendix 8: Convergence diagnosis map and density map of outcomes

**GA**


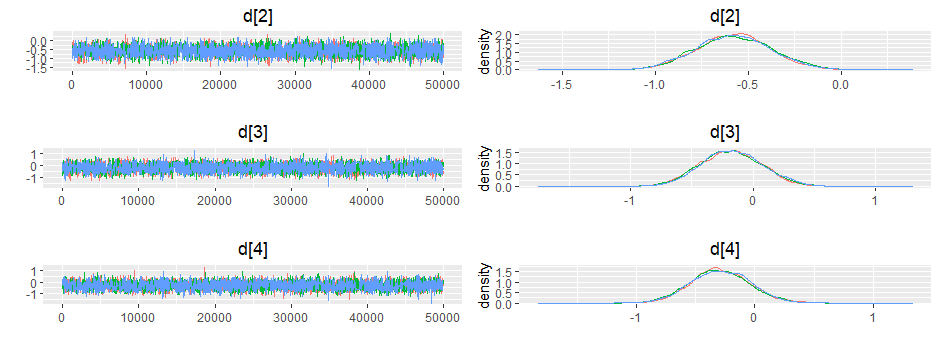


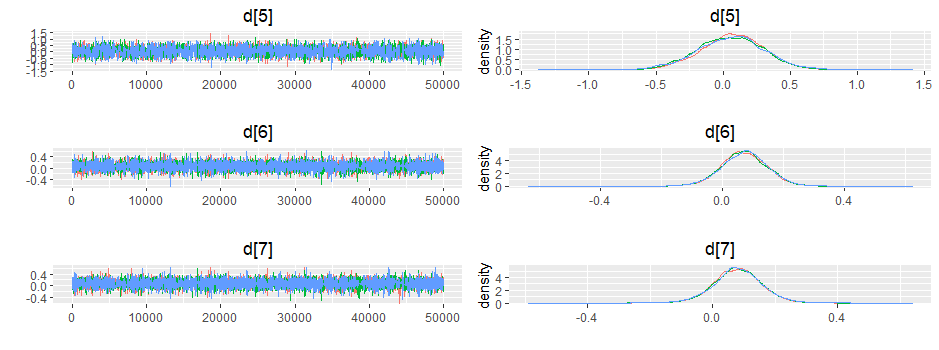


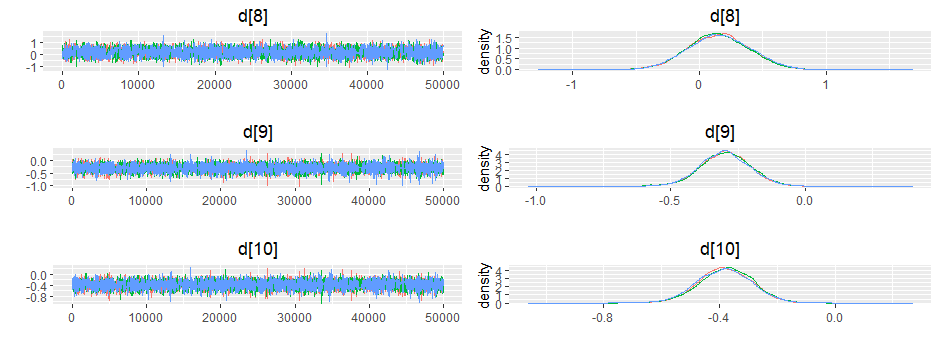


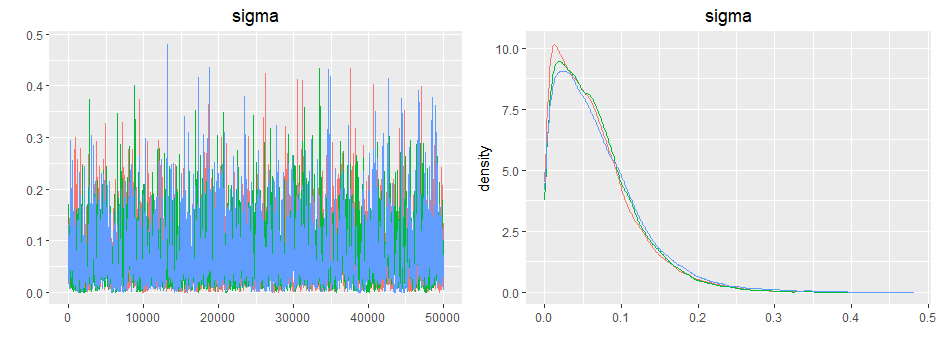


**BCVA**


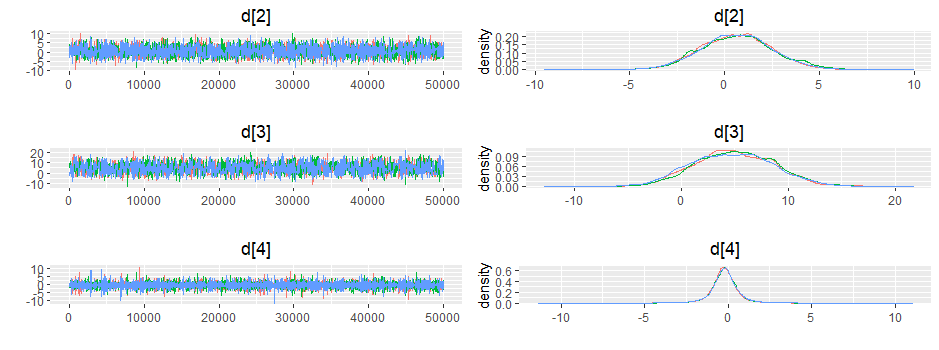

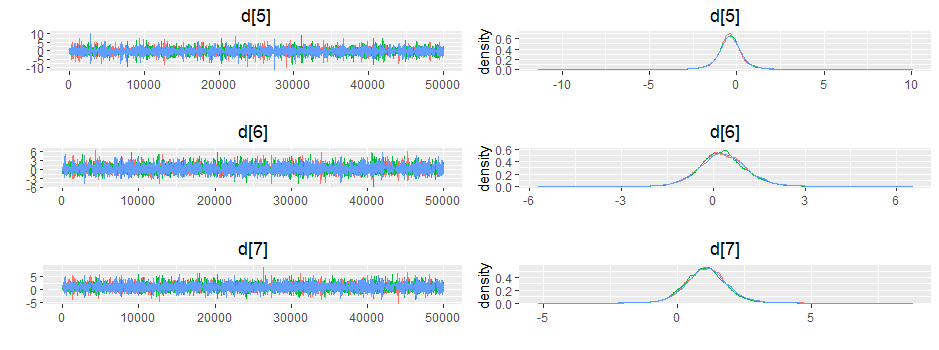

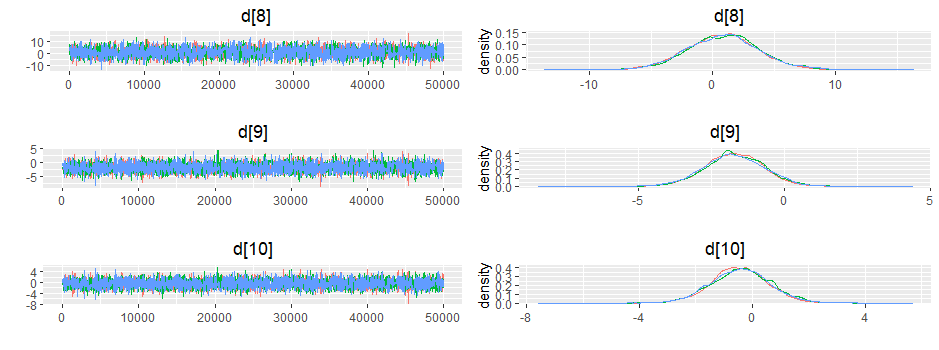

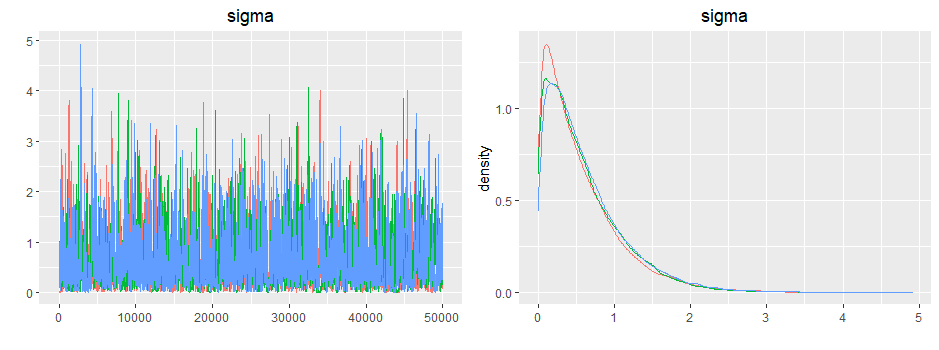


**SAE**


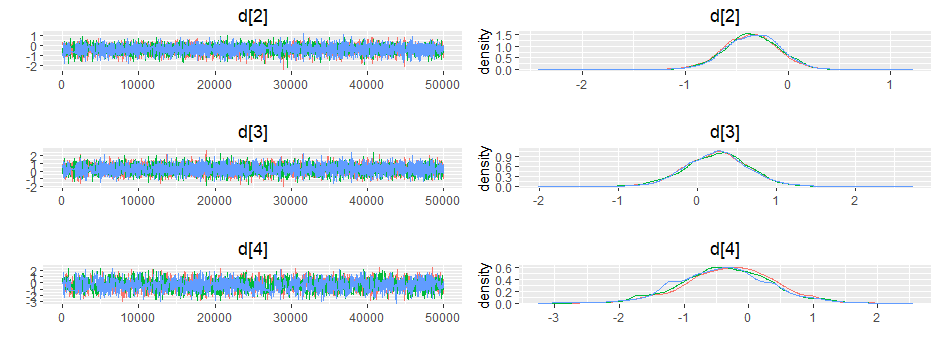

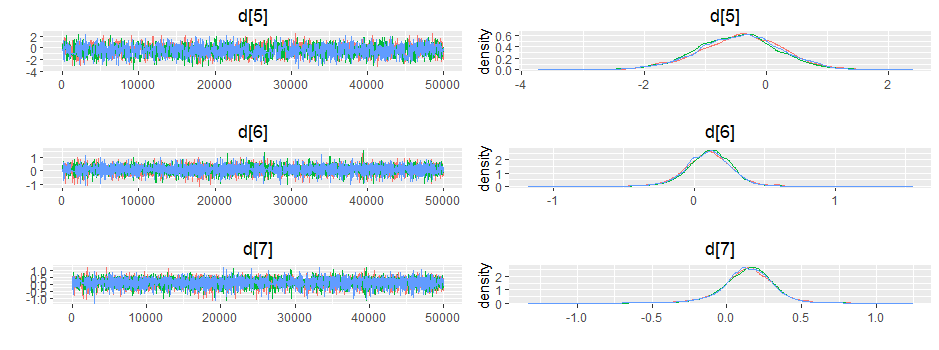

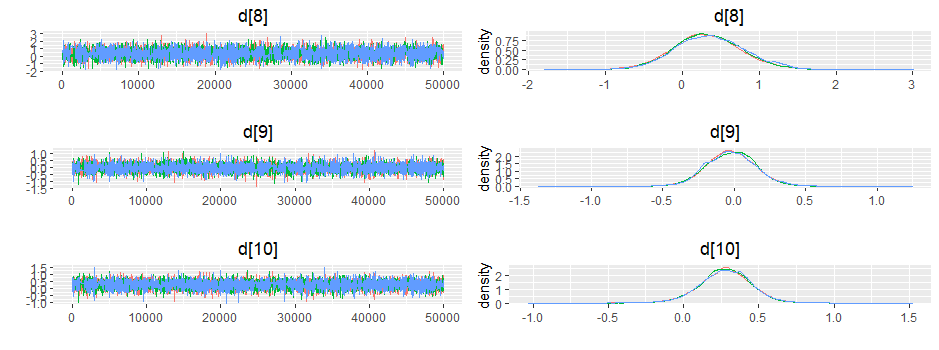

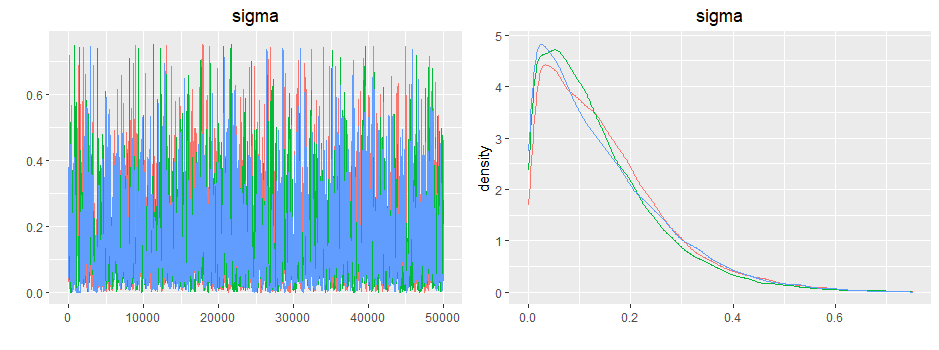


**MNV**


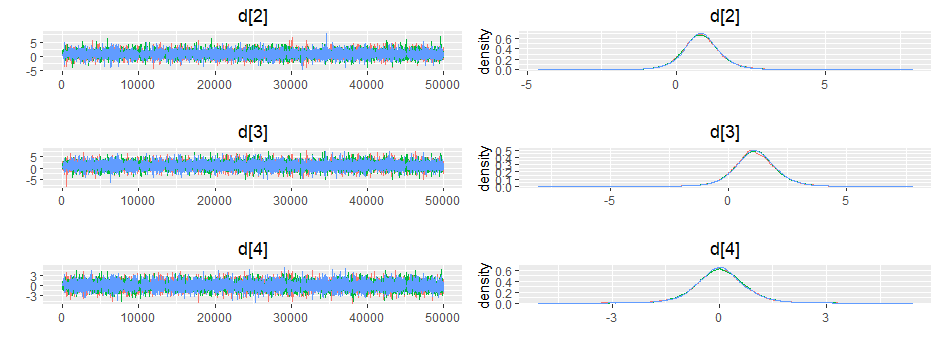

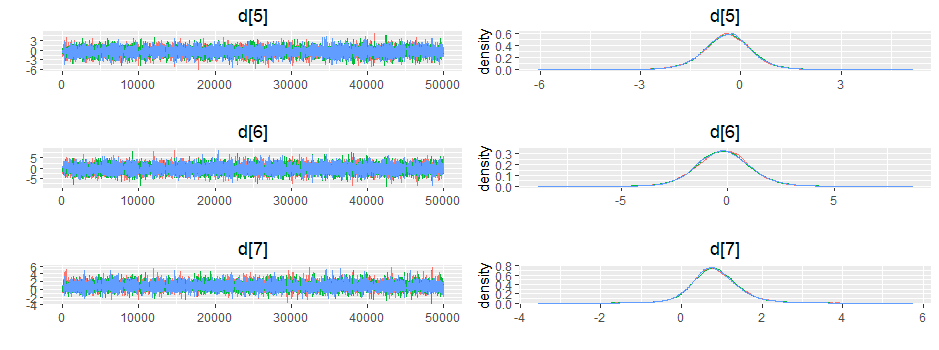

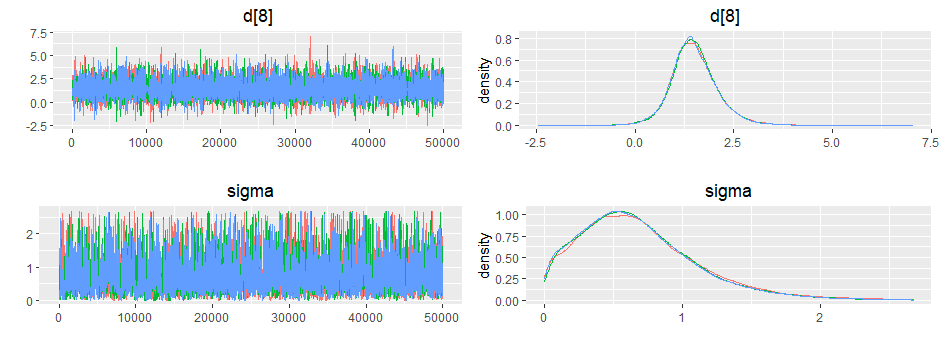


**Appendix 9: Comparison of the SAE between different treatments**


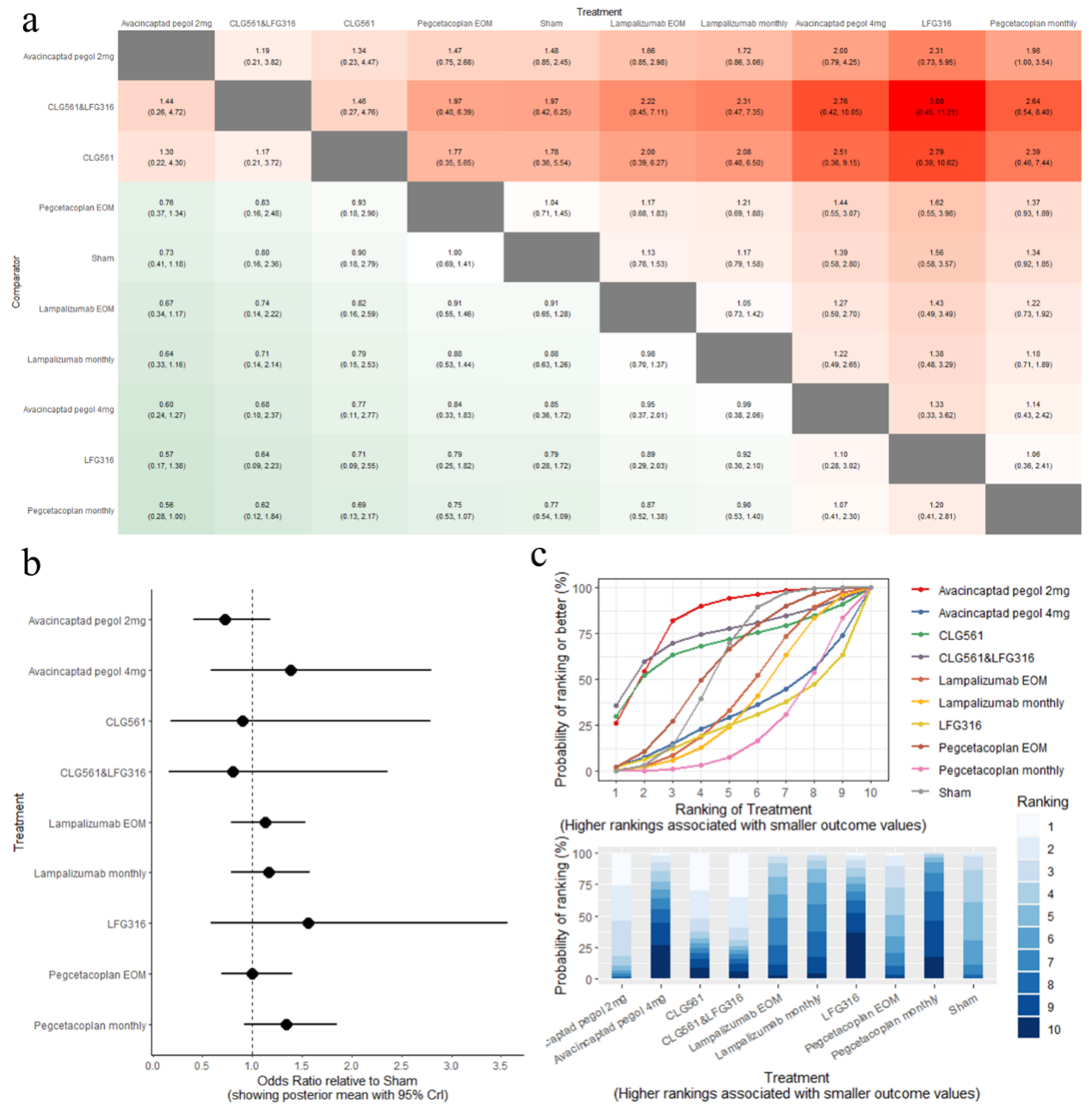

Supplement: Supplementary file 1 [file DataSheet1.docx]
